# An Improved Fuzzy Brain Emotional Learning Model Network Controller for Humanoid Robots

**DOI:** 10.3389/fnbot.2019.00002

**Published:** 2019-02-04

**Authors:** Wubing Fang, Fei Chao, Chih-Min Lin, Longzhi Yang, Changjing Shang, Changle Zhou

**Affiliations:** ^1^Cognitive Science Department, School of Information Science and Engineering, Xiamen University, Xiamen, China; ^2^Institute of Mathematics, Physics and Computer Science, Aberystwyth University, Aberystwyth, United Kingdom; ^3^Department of Electrical Engineering, Yuan Ze University, Tao-Yuan, Taiwan; ^4^Department of Computer and Information Sciences, Northumbria University, Newcastle upon Tyne, United Kingdom

**Keywords:** brain emotional learning network, humanoid robot control, Sliding mode control, neural network control, fuzzy neural network

## Abstract

The brain emotional learning (BEL) system was inspired by the biological amygdala-orbitofrontal model to mimic the high speed of the emotional learning mechanism in the mammalian brain, which has been successfully applied in many real-world applications. Despite of its success, such system often suffers from slow convergence for online humanoid robotic control. This paper presents an improved fuzzy BEL model (iFBEL) neural network by integrating a fuzzy neural network (FNN) to a conventional BEL, in an effort to better support humanoid robots. In particular, the system inputs are passed into a sensory and emotional channels that jointly produce the final outputs of the network. The non-linear approximation ability of the iFBEL is achieved by taking the BEL network as the emotional channel. The proposed iFBEL works with a robust controller in generating the hand and gait motion of a humanoid robot. The updating rules of the iFBEL-based controller are composed of two parts, including a sensory channel followed by the updating rules of the conventional BEL model, and the updating rules of the FNN and the robust controller which are derived from the “Lyapunov” function. The experiments on a three-joint robot manipulator and a six-joint biped robot demonstrated the superiority of the proposed system in reference to a conventional proportional-integral-derivative controller and a fuzzy cerebellar model articulation controller, based on the more accurate and faster control performance of the proposed iFBEL.

## 1. Introduction

The control of uncertain nonlinear systems with multiple inputs and outputs often presents a great challenge, and the robotic motion control is such a typical case. Robots, especially humanoid robots, are widely used in domestic, medical and other industrial areas (Liu et al., 2015; Li et al., 2017; Wu et al., 2018; Zhou et al., 2018). A humanoid robot must accurately control its two manipulators and two legs, in order to generate hand reaching/grasping motions and biped-leg walking gaits. Such crucial motion abilities allow humanoid robots to work in complicated, dangerous, and even poisonous environments with reduced labor costs, health implication, and other associated complications.

The Sliding Mode Control (SMC) proves to be an effective control method for uncertain nonlinear systems, especially for humanoid motion control. Once the state of the system reaches a sliding surface, the state will remain on that surface regardless of system uncertainties and external disturbances (Lin and Hsu, 2015). Yet, control input chattering, usually led by a combination of uncertainties from multiple pathways, is often not expected in humanoid robot systems when SMC is applied. It has been found in several studies that the collaboration of an artificial neural network with a SMC controller can enhance non-linear approximation ability in reducing the chattering effect (Boldbaatar and Lin, 2015).

A neural network with good non-linear learning abilities is therefore of great appeal to the SMC model. Note that an association between a stimulus and its emotional consequence in the amygdala of the mammalian brain was discovered by LeDoux (1992). The inspiration from the emotional consequence then led to the development of the brain emotional learning network (BEL) controller, which has a good nonlinear approximation capability. Such a neural network is comprised of a sensory neural network in simulating the orbitofrontal cortex of the brain, and an emotional neural network representing the amygdala cortex (LeDoux, 1992; Lotfi and Akbarzadeht, 2013). The sensory neural network is responsible for the major output of the controller, while the emotional neural network has an indirect impact on the sensory neural network. Despite of the effectiveness in uncertain non-linear control, most BEL networks face the dilemma of slow learning convergence leading to difficulty in on-line control of the multiple joints of a humanoid robot.

Fuzzy neural networks (FNN) are another popular choice for uncertain nonlinear control systems with reasonable non-linear approximation ability, due to their rapid learning convergence and simple structure which is particularly favorable for on-line humanoid robotic control (Rubio, 2012, 2018; Aguilar-Iban et al., 2018; Rubio et al., 2018). A typical FNN integrates a fuzzy inference system and a neural network (Pan et al., 2016; Meda-Campana et al., 2018). The weights of the network are usually updated by taking only the output errors of the FNN as the learning assessment. To achieve better performance for uncertain nonlinear systems, the FNN must also consider the overall performance of uncertain nonlinear systems when adjusting the control parameters, as reported in Zhao and Lin (2017). Therefore, the combination of the rapid convergence of FNN and the nonlinear mapping capability of BEL seems to be a good idea for controlling humanoid robots.

We believe that the chattering effect of the SMC model is a very challenging issue. Although, many existing algorithms had been developed to deal with the chatting; the artificial neural network still plays an important role in the control of uncertain nonlinear system with multiple inputs and outputs. In addition, FNN is good at rapid convergence and BEL can ideally increase the network's nonlinear mapping capability. Therefore, we focused on a combined neural network to deal with the chattering problem. Based on these considerations, this paper proposes an improved brain emotional learning model network (iFBEL) for a humanoid robot controller, in an effort to achieve better human-like control performance with the support of more nonlinear approximation capabilities. The proposed iFBEL is comprised of two components, with one built from a conventional BEL and the other created by an FNN; and the resulted iFBEL thus enjoys the advantages of both sub-systems. The iFBEL works with a robust controller to replace the ideal sliding mode controller for better system performance. To ensure the convergence and robustness, the adaptive laws of the FNN and the robust controller are derived from the Lyapunov function. The iFBEL was validated and evaluated on a robot with a three-joint manipulator and a biped-leg system, although applications in other control fields can be readily identified. The experimental results demonstrate competitive performance of the proposed systems in dynamic humanoid robotic control.

The reminder of this paper is organized as follows: section 2 introduces a group of uncertain nonlinear systems controlled by a sliding mode controller. Section 3 reports the proposed improved fuzzy brain emotional learning model neural network. Section 4 describes the implementations of the network controller and the updating rules. Section 5 shows the experimental results and compares the performances with the conventional proportional-integral-derivative (PID) controller and the fuzzy cerebellar model articulation controller (FCMAC). Section 7 concludes the paper and points out future work.

## 2. Humanoid Robot Control by Sliding Mode Controller

In order to understand the proposed network-based control system and realize the importance of the proposed neural network, this section introduces a typical uncertain nonlinear system controlled by a sliding mode controller as the work's background.

A humanoid robot needs to control multi-joints. Without loss of generality, consider a class of *n*th-order uncertain nonlinear systems with *m*th-order input and output states expressed in the following form:

(1) $$x^{\left(n\right)}(t)=f(\underline{x}(t))+G(\underline{x}(t))u(t)+d(t),$$

where *x*(*t*) = [*x*^(*n*−1)^(*t*) … *ẋ*(*t*) *x*(*t*)] ∈ ℜ^*m*×*n*^ is the system state vector, $u\left(t\right)={\left[u_{1}\left(t\right),u_{2}\left(t\right),\ldots ,u_{m}\left(t\right)\right]}^{T}\in {ℜ}^{m}$ is the control input vector, *f*(*x*(*t*)) ∈ ℜ^*m*^ is an unknown, but bounded, smooth nonlinear function, *G*(*x*(*t*)) ∈ ℜ^*m*×*m*^ is an unknown, but bounded, gain matrix, and $d\left(t\right)={\left[d_{1}\left(t\right),d_{2}\left(t\right),\ldots ,d_{m}\left(t\right)\right]}^{T}\in {ℜ}^{m}$ is an external bounded disturbance.

The nominal model of such a nonlinear system can be defined as

(2) $$\begin{array}{r} x^{\left(n\right)}\left(t\right)=f_{n}\left(\underline{x}\left(t\right)\right)+G_{n}u\left(t\right), \end{array}$$

where *f*_*n*_(*x*(*t*)) is the nominal function of *f*(*x*(*t*)), and $G_{n}=diag\left[g_{n_{1}}\ldots g_{n_{m}}\right]\in {ℜ}^{m\times m}$ is the nominal function of *G*(*x*(*t*)), with *g*_*n*_*i*__ being nominal gain constants, for *i* = 1, 2, …, *m*. Assume that *g*_*n*_*i*__ > 0 for the existence of $G_{n}^{-1}$, Equation 1 can be represented as:

(3) $$\begin{array}{l} \begin{array}{l} x^{\left(n\right)}\left(t\right)=f_{n}\left(\underline{x}\left(t\right)\right)+△f\left(\underline{x}\left(t\right)\right)+G_{n}u\left(t\right)+△G\left(\underline{x}\left(t\right)\right)u\left(t\right)+d\left(t\right)\\ =f_{n}\left(\underline{x}\left(t\right)\right)+G_{n}u\left(t\right)+l\left(\underline{x}\left(t\right),t\right), \end{array} \end{array}$$

where *l*(*x*(*t*), *t*) = △*f*(*x*(*t*)) + △*G*(*x*(*t*))*u*(*t*) + *d*(*t*) is the lumped uncertainties and external disturbances. Let ${\underline{x}}_{d}\left(t\right)={\left[x_{d}^{\left(n-1\right)T}\left(t\right),\ldots ,{ẋ}_{d}^{T}\left(t\right),x_{d}^{T}\left(t\right)\right]}^{T}\in {ℜ}^{m\times n}$ be a desired trajectory in which the state of the system is tracked. The tracking error vector is defined as:

$$\begin{array}{l} \underline{e}\left(t\right)=\left[\begin{array}{ccccc} e^{\left(n-1\right)}\left(t\right) & e^{\left(n-2\right)}\left(t\right) & \ldots & ė\left(t\right) & e\left(t\right) \end{array}\right]\in {ℜ}^{mn}, \end{array}$$

where *e*(*t*) = *x*_*d*_(*t*) − *x*(*t*) is the tracking error.

An ideal sliding surface can be defined as

(4) $$\begin{array}{ll} s\left(\underline{e}\left(t\right)\right)= & \left(\begin{array}{c} s_{1}\\ s_{2}\\ \vdots \\ s_{m} \end{array}\right)\\ =\left[\begin{array}{ccc} e_{1}^{\left(n-1\right)}\left(t\right)+ & \lambda_{11}e_{1}^{\left(n-2\right)}\left(t\right)+\cdots + & \lambda_{n1}\int_{0}^{T}e_{1}\left(t\right)dt\\ e_{2}^{\left(n-1\right)}\left(t\right)+ & \lambda_{12}e_{2}^{\left(n-2\right)}\left(t\right)+\cdots + & \lambda_{n2}\int_{0}^{T}e_{2}\left(t\right)dt\\ \vdots & \vdots & \vdots \\ e_{m}^{\left(n-1\right)}\left(t\right)+ & \lambda_{1m}e_{m}^{\left(n-2\right)}\left(t\right)+\cdots + & \lambda_{nm}\int_{0}^{T}e_{m}\left(t\right)dt \end{array}\right]\\ =\left[\begin{array}{ccc} 1 & \lambda_{11} & \lambda_{n1}\\ \ddots & \ddots & \ddots \\ 1 & \lambda_{1m} & \lambda_{nm} \end{array}\right]\left[\begin{array}{c} \underline{e}\left(t\right)\\ \int_{0}^{T}e\left(t\right)dt \end{array}\right] \end{array}$$

$$\begin{array}{r} =\bar{K}\left[\begin{array}{c} \underline{e}\left(t\right)\\ \int_{0}^{T}e\left(t\right)dt \end{array}\right], \end{array}$$

where $\bar{K}=\left[I,K\right]=\left[\begin{array}{cccc} I & \lambda_{1}I & \ldots & \lambda_{n}I \end{array}\right]\in {ℜ}^{m\times \left(m+1\right)n}$. All $\lambda_{j}={\left[\lambda_{1j}\ldots \lambda_{nj}\right]}^{T}\in {ℜ}^{n}$ are roots of the equation: $q^{n}+\lambda_{1}q^{n-1}+\cdots +\lambda_{n-1}q+\lambda_{n}=0$ in which *q* is the Laplace operator and is in the open left half-plane. The time derivative of Equation 4 leads to the following:

(5) $$\begin{array}{l} \begin{array}{l} ṡ\left(\underline{e}\left(t\right)\right)=\bar{K}\left[\begin{array}{c} \underline{ė}\left(t\right)\\ e\left(t\right) \end{array}\right]=\bar{K}\left[\begin{array}{c} e^{\left(n\right)}\left(t\right)\\ \underline{e}\left(t\right) \end{array}\right]\\ =e^{\left(n\right)}\left(t\right)+K\underline{e}\left(t\right)=x_{d}^{\left(n\right)}\left(t\right)-x^{\left(n\right)}\left(t\right)+K\underline{e}\left(t\right)\\ =x_{d}^{\left(n\right)}\left(t\right)-f_{n}\left(\underline{x}\left(t\right)\right)-G_{n}u\left(t\right)-l\left(\underline{x}\left(t\right),t\right)+K\underline{e}\left(t\right), \end{array} \end{array}$$

where *ė*(*t*) = [*e*^(*n*)^(*t*)*e*^(*n*−1)^(*t*) … *ė*(*t*)].

For the existence and reachability of this sliding surface, the control law of system is satisfied by the following inequation:

(6) $$\begin{array}{l} \frac{1}{2}\frac{d}{dt}\left(s_{i}^{2}\right)\leq -\overset{m}{\underset{i=1}{\sum }}\sigma_{i}|s_{i}| \end{array}$$

for σ_*i*_ > 0, *i* = 1, 2, …, *m*.

Taking 5 into 6, yields

(7) $$\begin{array}{l} s^{T}(\underline{e}(t))\dot{s}(\underline{e}(t))=s^{T}(\underline{e}(t))[x_{d}^{\left(n\right)}(t)-f_{n}(\underline{x}(t))-G_{n}u(t)-l(\underline{x}(t),t)\\ \;+\;K\underline{e}(t)]\leq -\sum_{i=1}^{m}\sigma_{i}|s_{i}| \end{array}$$

If the dynamic and the lumped uncertainty of the system are known exactly, the ideal sliding mode controller is designed as:

(8) $$\begin{array}{l} u_{ISMC}=G_{n}^{-1}\left[x_{d}^{\left(n\right)}\left(t\right)-f_{n}\left(\underline{x}\left(t\right)\right)-l\left(\underline{x}\left(t\right),t\right)+K\underline{e}\left(t\right)+\sigma sgn\left(s\left(\underline{e}\left(t\right)\right)\right)\right], \end{array}$$

where *sgn* is a sign function and *G*_*n*_ is a positive define matrix. However, it is difficult to obtain the dynamical functions of most nonlinear systems, and the lumped uncertainty is always unmeasurable. Therefore, the ideal sliding mode controller is unobtainable.

## 3. The Proposed iFBEL Network

**Figure 1:**
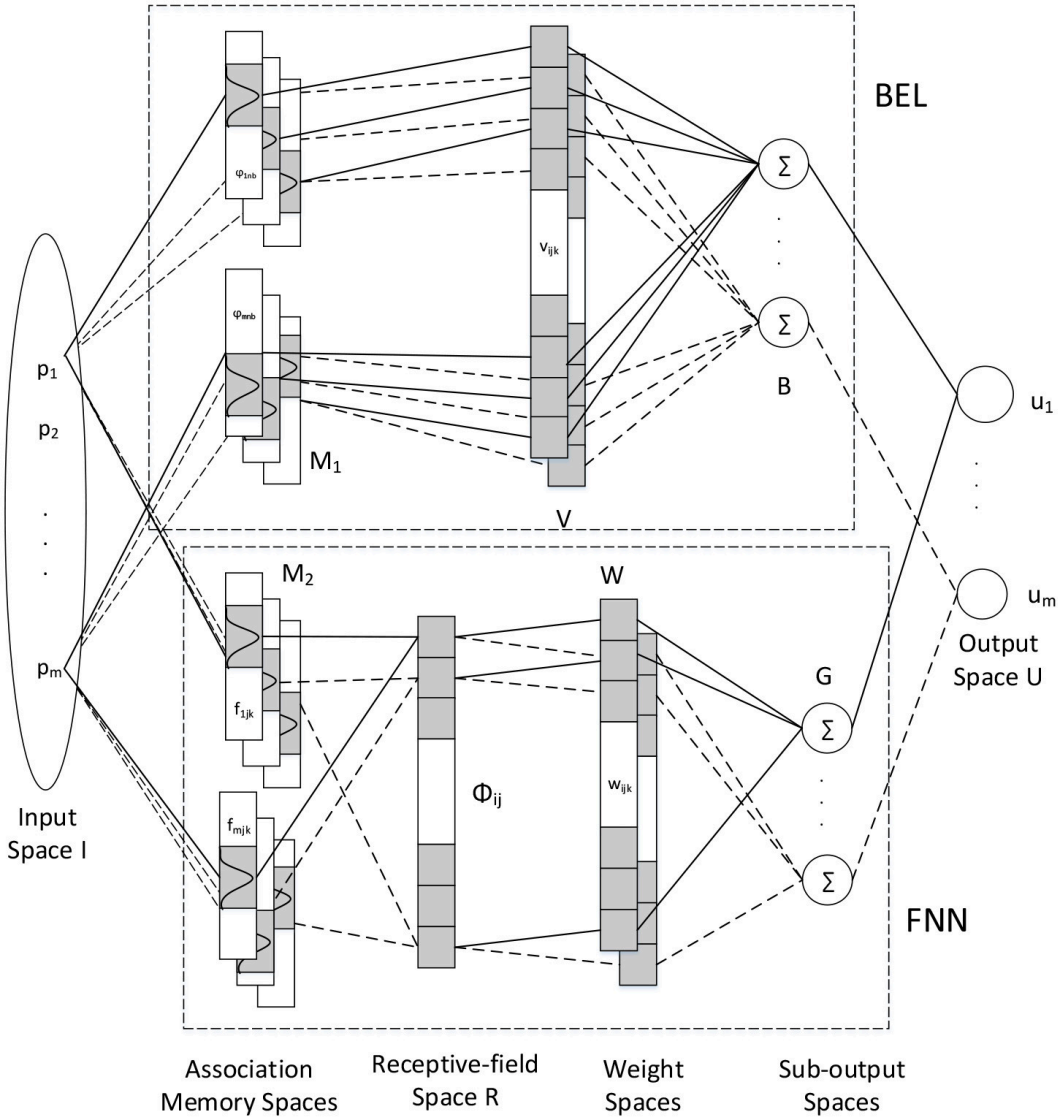
The configuration of iFBEL.

The configuration of the proposed iFBEL is depicted in Figure 1, consisting of an BEL and the FNN in addition to the input and output spaces. The outputs of this network are *u*_*i*_ = *b*_*i*_ − *g*_*i*_ for *i* = 1, 2, …, *m*, in which, *b*_*i*_ are the outputs of the the BEL and *g*_*i*_ are the outputs of the FNN. The BEL network is comprised of the input space *I*, the association memory space *M*_1_, the weight memory space *V*, and the sub-output space *B*. The FNN shares the same input space with the BEL, and it also includes the association memory space *M*_2_, the receptive-field space *R*, the weight memory space *W*, and the sub-output space *G*. In particular, the FNN channel of iFBEL also contains a set of fuzzy reference rules (Lee, 1990) as represented as follows:

(9) $$\begin{array}{l} \begin{array}{l} R^{\lambda }:\text{If}p_{1}\text{is}\phi_{1jk}\text{and}p_{2}\text{is}\phi_{2jk},\ldots ,p_{m}\text{is}\phi_{mjk}\text{then}\\ g_{jk}=\omega_{jk}\text{for}j=1,2,\ldots ,n_{f}.\;k=1,2,\ldots ,n_{k}.\;\lambda =1,2,\ldots ,n_{l}, \end{array} \end{array}$$

where *n*_*f*_ is the number of layers for each *m* input dimensions with each layer including *n*_*k*_ blocks and *n*_*l*_ = *n*_*f*_*n*_*k*_ referring to the number of fuzzy rules, and ϕ_*ijk*_ represents the fuzzy set for *i*th input, *j*th layer and *k*th block; each fuzzy set's member function is implemented by the Gaussian function; ω_*jk*_ is the output weight in the consequent part; and *g*_*jk*_ is the rule's output. Note that: each fuzzy set's member function can be defined as rectangular, triangular or any continuously bounded function e.g., Gaussian or B-spline; in order to easily implement the iFBEL with the better non-linear approximation ability, the Gaussian function is adopted.

**Figure 2:**
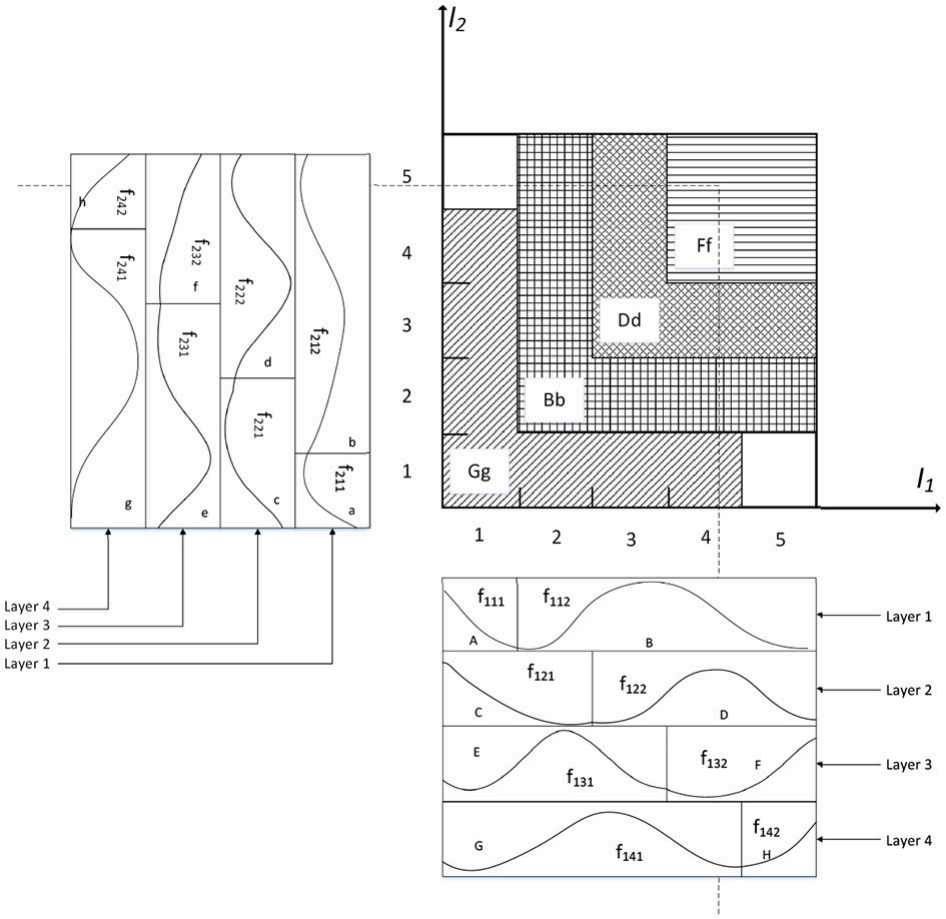
Organization of an example 2-D FNN.

The aforementioned “spaces” are detailed as follows:

1. *Input Space I*: $p={\left[p_{1},p_{2},\ldots ,p_{m}\right]}^{T}\in {ℜ}^{m}$ is an input vector which is quantized into discrete regions (elements), where *m* is the number of input state variables. The number of elements, *n*_*e*_, is termed as a resolution. *p* is delivered to the BEL and the FNN simultaneously as their inputs.
2. *Association Memory Spaces M*_1_ and *M*_2_: Several elements are combined as a block; the number of blocks, *n*_*b*_ and *n*_*f*_ for the BEL and the FNN respectively, must be equal or greater than two. The association memory space of the BEL has *n*_*a*_(= *m* × *n*_*b*_) components, while that of the FNN has *n*_*c*_(= *m* × *n*_*f*_) components. Every component is represented as a Gaussian basis function; let φ denote a component for the BEL and *f* for the FNN:
  (10) $$\begin{array}{l} \varphi_{ij}=exp\left[-\frac{{\left(p_{i}-y_{ij}\right)}^{2}}{z_{ij}^{2}}\right] \end{array}$$
  where *i* = 1, 2, …, *m*, *j* = 1, 2, …, *n*_*b*_, and *y*_*ij*_ and *z*_*ij*_ are the means and variances, respectively; and
  (11) $$\begin{array}{l} f_{ijk}=exp\left[-\frac{{\left(p_{i}-c_{ijk}\right)}^{2}}{v_{ijk}^{2}}\right] \end{array}$$
  where *i* = 1, 2, …, *m*, *j* = 1, 2, …, *n*_*f*_, *k* = 1, 2, …, *n*_*k*_, and *c*_*ijk*_ and *v*_*ijk*_ are the means and variances, respectively.
  The block matrix of the BEL is defined as:
  (12) $$\begin{array}{l} \Gamma ={\left[\begin{array}{cccccccccc} \varphi_{11} & \ldots & \varphi_{1n_{b}} & \varphi_{21} & \ldots & \varphi_{2n_{b}} & \ldots & \varphi_{m1} & \ldots & \varphi_{mn_{b}} \end{array}\right]}^{T}\in {ℜ}^{mn_{b}}. \end{array}$$
3. *Receptive-field Space R for FNN*: Every cell in this space is the product of the corresponding components of the association memory space *M*_2_, which is defined as:
  (13) $$\begin{array}{l} \phi_{jk}=\overset{m}{\underset{i=1}{\prod }}f_{ijk}=\overset{m}{\underset{i=1}{\prod }}exp\left[-\frac{{\left(p_{i}-c_{ijk}\right)}^{2}}{v_{ijk}^{2}}\right]=exp\left[-\overset{m}{\underset{i=1}{\sum }}\frac{{\left(p_{i}-c_{ijk}\right)}^{2}}{v_{ijk}^{2}}\right], \end{array}$$
  where *j* = 1, 2, …, *n*_*f*_, and *k* = 1, 2, …, *n*_*k*_. An example of the FNN with two input variables is shown in Figure 2, which has 4 layers (*n*_*f*_ = 4) for every input variable and 2 blocks (*n*_*k*_ = 2) for each layer. And *n*_*l*_ = *n*_*f*_*n*_*k*_ is the number of receptive fields, such as *Aa, Bb*, …; ϕ_*jk*_ is associated with the *j*th layer and the *k*th block in the fuzzy rule as expressed in Equation 9. The block matrix of the FNN is defined as:
  (14) $$\begin{array}{l} \Phi ={\left[\begin{array}{cccccccccc} \phi_{11} & \ldots & \phi_{1n_{k}} & \phi_{21} & \ldots & \phi_{2n_{k}} & \ldots & \phi_{n_{f}1} & \ldots & \phi_{n_{f}n_{k}} \end{array}\right]}^{T}\in {ℜ}^{n_{f}n_{k}}. \end{array}$$
4. *Weight Memory Spaces V* and *W* : ν_*ijk*_ is the weight of the *i*th output, *j*th input, and *k*th block of the BEL; and ω_*ijk*_ is the weight of the *i*th output, *j*th layer, *k*th block of the FNN:
  $$\begin{array}{l} V=\left[\begin{array}{cccc} \nu_{1jk} & \nu_{2jk} & \ldots & \nu_{mjk} \end{array}\right]\\ =\left[\begin{array}{cccc} \nu_{111} & \nu_{211} & \ldots & \nu_{m11}\\ \vdots & \vdots & \vdots \\ \nu_{11n_{b}} & \nu_{21n_{b}} & \ldots & \nu_{m1n_{b}}\\ \nu_{121} & \nu_{221} & \ldots & \nu_{m21}\\ \vdots & \vdots & \vdots \\ \nu_{12n_{b}} & \nu_{22n_{b}} & \ldots & \nu_{m2n_{b}}\\ \vdots & \vdots & \vdots \\ \nu_{1m1} & \nu_{2m1} & \ldots & \nu_{mm1}\\ \vdots & \vdots & \vdots \\ \nu_{1mn_{b}} & \nu_{2mn_{b}} & \ldots & \nu_{mmn_{b}} \end{array}\right]\in {ℜ}^{mn_{b}\times m}\\ W=\left[\begin{array}{cccc} \omega_{1jk} & \omega_{2jk} & \ldots & \omega_{mjk} \end{array}\right] \end{array}$$
  (15) $$\begin{array}{l} =\left[\begin{array}{cccc} \omega_{111} & \omega_{211} & \ldots & \omega_{m11}\\ \vdots & \vdots & \vdots \\ \omega_{11n_{f}} & \omega_{21n_{f}} & \ldots & \omega_{m1n_{f}}\\ \omega_{121} & \omega_{221} & \ldots & \omega_{m21}\\ \vdots & \vdots & \vdots \\ \omega_{12n_{f}} & \omega_{22n_{f}} & \ldots & \omega_{m2n_{f}}\\ \vdots & \vdots & \vdots \\ \omega_{1n_{k}1} & \omega_{2n_{k}1} & \ldots & \omega_{mn_{k}1}\\ \vdots & \vdots & \vdots \\ \omega_{1n_{k}n_{f}} & \omega_{2n_{k}n_{f}} & \ldots & \omega_{mn_{k}n_{f}} \end{array}\right]\in {ℜ}^{n_{f}n_{k}\times m}. \end{array}$$
5. *Sub-output Space B* and *G*: The *i*th output (*b*_*i*_) and the output vector (*b*) of the BEL, and the *i*th output (*g*_*i*_) and the output vector (*g*) of the FNN are represented as follows:
  (16) $$\begin{array}{l} b_{i}=\overset{m}{\underset{j=1}{\sum }}\overset{n_{b}}{\underset{k=1}{\sum }}\nu_{ijk}\varphi_{jk}, \end{array}$$
  (17) $$\begin{array}{l} b={\left[\begin{array}{cccc} b_{1} & b_{2} & \ldots & b_{m} \end{array}\right]}^{T}=V^{T}\cdot \Gamma , \end{array}$$
  (18) $$\begin{array}{l} g_{i}=\overset{n_{f}}{\underset{j=1}{\sum }}\overset{n_{k}}{\underset{k=1}{\sum }}\omega_{ijk}\phi_{jk}, \end{array}$$
  (19) $$\begin{array}{l} g={\left[\begin{array}{cccc} g_{1} & g_{2} & \ldots & g_{m} \end{array}\right]}^{T}=W^{T}\cdot \Phi . \end{array}$$
6. *Output Space U*: The output of the proposed iFBEL is the combination of the outputs of the BEL and the FNN, in which the BEL works as a primary controller and the FNN as an emotion controller:
  (20) $$\begin{array}{l} u_{i}=b_{i}-g_{i}=\overset{m}{\underset{j=1}{\sum }}\overset{n_{b}}{\underset{k=1}{\sum }}\nu_{ijk}\varphi_{jk}-\overset{n_{f}}{\underset{j=1}{\sum }}\overset{n_{k}}{\underset{k=1}{\sum }}\omega_{ijk}\phi_{jk}, \end{array}$$
  (21) $$\begin{array}{l} u=b-g=V^{T}\cdot \Gamma -W^{T}\cdot \Phi . \end{array}$$

## 4. iFBEL-based Controller

**Figure 3:**
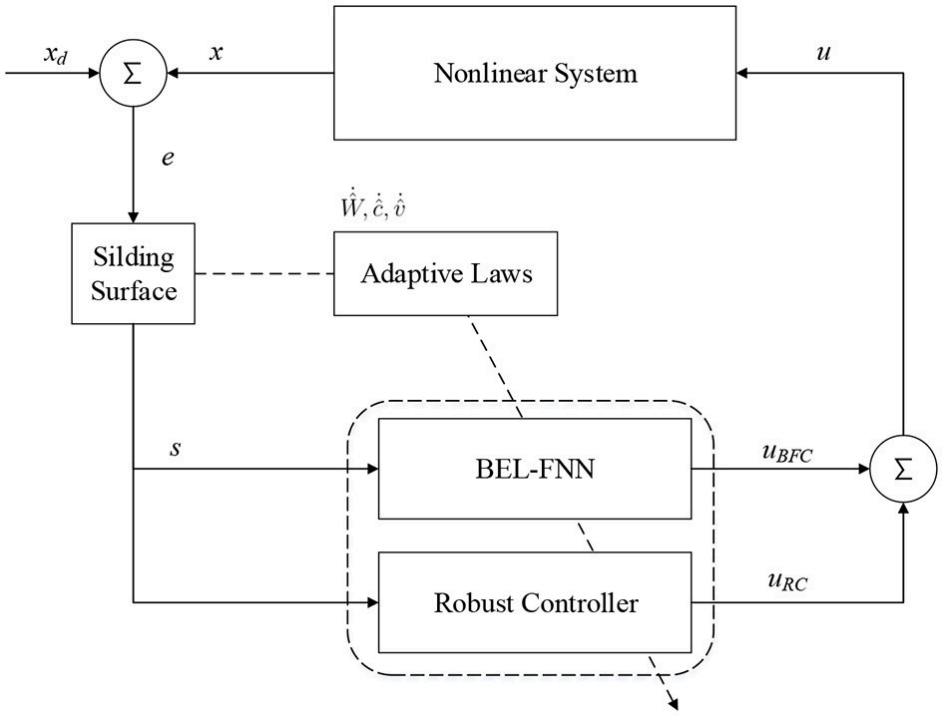
Design of control system.

The proposed intelligent controller, consisting of a sliding surface, an iFBEL network, and a robust controller, is shown in Figure 3. The iFBEL network and robust controller collaborate to imitate an ideal sliding mode controller. The updating rules of the BEL mechanism of the iFBEL network are followed by the brain emotional learning algorithm (Chung and Lin, 2015; Lin and Chung, 2015); and the adaptive laws of the FNN mechanism and robust controller are derived from the Lyapunov function. Besides, to ensure robust tracking performance.

The updating rules are detailed as follows. Subtracting 8 into 5, yields:

(22) $$\begin{array}{l} ṡ\left(\underline{e}\left(t\right)\right)=G_{n}\left[u_{ISMC}-u\right]-\sigma sgn\left[s\left(\underline{e}\left(t\right)\right)\right]. \end{array}$$

Assume that an optimal iFBEL $u_{BFC}^{*}$ exists in the ideal sliding model controller, *u*_*ISMC*_, and that ϵ is a minimum error vector; thus, the weight matrixes of $u_{BFC}^{*}$ are represented as V^*^ and W^*^ for the BEL and the FNN, respectively. Then, the output of the optimal sliding model controller is:

(23) $$\begin{array}{l} \begin{array}{l} u_{ISMC}=u_{BFC}^{*}+\epsilon ={\left(u_{BEL}-u_{FNN}\right)}^{*}+\epsilon \\ ={\left(V^{T}\Gamma -W^{T}\Phi \right)}^{*}+\epsilon =V^{*T}\hat{\Gamma }-W^{*T}\Phi^{*}+\epsilon , \end{array} \end{array}$$

where *u*_*BEL*_ and *u*_*FNN*_ are the outputs of the BEL and the FNN respectively, and Φ^*^ and $\hat{\Gamma }$ are the optimal matrix and estimated matrix of Φ and Γ respectively. The output of the proposed iFBEL controller is defined by:

(24) $$\begin{array}{l} u=u_{BFC}+u_{RC}={\hat{V}}^{T}\hat{\Gamma }-{Ŵ}^{T}\hat{\Phi }+u_{RC}, \end{array}$$

where *u*_*RC*_ is the output of the robust controller, and $\hat{V},Ŵ,\hat{\Phi }$ are the estimated matrices of V^*^, W^*^, Φ^*^ respectively.

Taking 23 and 24 into 22, the following can be obtained:

$$\begin{array}{lll} ṡ\left(\underline{e}\left(t\right)\right) & = & G_{n}\left[V^{*T}\hat{\Gamma }-W^{*T}\Phi^{*}+\epsilon -{\hat{V}}^{T}\hat{\Gamma }+{Ŵ}^{T}\hat{\Phi }-u_{RC}\right] \end{array}$$

(25) $$\begin{array}{l} -\sigma sgn\left[s\left(\underline{e}\left(t\right)\right)\right] \end{array}$$

$$\begin{array}{ll} = & G_{n}\left[{Ṽ}^{T}\hat{\Gamma }-{\tilde{W}}^{T}\Phi^{*}-{Ŵ}^{T}\tilde{\Phi }+\epsilon -u_{RC}\right]-\sigma sgn\left[s\left(\underline{e}\left(t\right)\right)\right], \end{array}$$

where $\tilde{\Phi }=\Phi^{*}-\hat{\Phi }$, and $Ṽ=V^{*}-\hat{V}$. A partially linear form of the receptive-field basis function vector $\tilde{\Phi }$ in the Taylor series is:

$$\begin{array}{lll} \tilde{\Phi } & = & \left(\begin{array}{c} \tilde{\phi_{1}}\\ \vdots \\ \tilde{\phi_{n_{d}}} \end{array}\right)=\left(\begin{array}{c} {\left(\frac{\partial \phi_{1}}{\partial c}\right)}^{T}\\ \vdots \\ {\left(\frac{\partial \phi_{n_{d}}}{\partial c}\right)}^{T} \end{array}\right){|}_{c=ĉ}\left(c^{*}-ĉ\right)+\left(\begin{array}{c} {\left(\frac{\partial \phi_{1}}{\partial v}\right)}^{T}\\ \vdots \\ {\left(\frac{\partial \phi_{n_{d}}}{\partial v}\right)}^{T} \end{array}\right){|}_{v=\hat{v}}\left(v^{*}-\hat{v}\right)+\beta \end{array}$$

(26) $$\begin{array}{ll} = & \Phi_{c}\tilde{c}+\Phi_{v}ṽ+\beta , \end{array}$$

where Φ_*c*_ and Φ_*v*_ are defined by:

$$\begin{array}{l} \Phi_{c}={\left[\frac{\partial \phi_{1}}{\partial c},\ldots ,\frac{\partial \phi_{n_{d}}}{\partial c}\right]}^{T}{|}_{c=ĉ}\in {ℜ}^{n_{d}\times n_{f}n_{d}}\\ \Phi_{v}={\left[\frac{\partial \phi_{1}}{\partial v},\ldots ,\frac{\partial \phi_{n_{d}}}{\partial v}\right]}^{T}{|}_{v=\hat{v}}\in {ℜ}^{n_{d}\times n_{f}n_{d}}, \end{array}$$

where $\tilde{c}=c^{*}-ĉ,ṽ=v^{*}-\hat{v}$, β is a higher-order vector.

Rewriting 26 with $\tilde{\Phi }=\Phi^{*}-\hat{\Phi }$, yields:

(27) $$\begin{array}{l} \Phi^{*}=\hat{\Phi }+\tilde{\Phi }=\hat{\Phi }+\Phi_{c}\tilde{c}+\Phi_{v}ṽ+\beta . \end{array}$$

Substituting 27 to 25, yields:

(28) $$\begin{array}{l} \dot{s}(\underline{e}(t))=G_{n}[{\tilde{V}}^{T}\hat{\Gamma }-{\tilde{W}}^{T}(\hat{\Phi }+\Phi_{c}\tilde{c}+\Phi_{v}\tilde{v}+\beta )-{\hat{W}}^{T}\\ \;(\Phi_{c}\tilde{c}+\Phi_{v}\tilde{v}+\beta )+\epsilon -u_{RC}]-\sigma sgn[s(\underline{e}(t))]\\ \;=\;G_{n}[{\tilde{V}}^{T}\hat{\Gamma }-{\tilde{W}}^{T}\hat{\Phi }-{\hat{W}}^{T}(\Phi_{c}\tilde{c}+\Phi_{v}\tilde{v})-u_{RC}+\omega ]\\ \;-\sigma sgn[s(\underline{e}(t))], \end{array}$$

where $\omega =W^{*T}\beta +\tilde{W}\left(\Phi_{c}\tilde{c}+\Phi_{v}ṽ\right)+\epsilon$ is a combined error of the FNN, and $Ṽ=V^{*}-\hat{V}={\left[\tilde{\nu_{1}},\tilde{\nu_{2}},\ldots ,\tilde{\nu_{m}}\right]}^{T}\in {ℜ}^{m\times mn_{b}}$ is an approximation error weight matrix of the BEL. Consider a *H*_∞_ tracking performance (Chen et al., 2015) for the existence of ω and Ṽ as:

$$\begin{array}{l} \sum_{i=1}^{m}\int_{0}^{T}s_{i}^{2}\left(t\right)dt\leq s^{T}\left(0\right)G_{n}^{-1}s\left(0\right)+tr\left[{\tilde{W}}^{T}\left(0\right)\eta_{W}^{-1}\tilde{W}\left(0\right)\right]\\ +\;{\tilde{c}}^{T}\left(0\right)\eta_{c}^{-1}\tilde{c}\left(0\right)+{ṽ}^{T}\left(0\right)\eta_{v}^{-1}ṽ\left(0\right)+\overset{m}{\underset{i=1}{\sum }}\lambda_{i}^{2} \end{array}$$

(29) $$\begin{array}{l} \int_{0}^{T}\omega_{i}^{2}\left(t\right)dt+\overset{m}{\underset{i=1}{\sum }}\int_{0}^{T}{\tilde{\nu }}_{i}^{2}\left(t\right)dt, \end{array}$$

where η_*W*_, η_*c*_, η_*v*_ are diagonal positive constant learning-rate matrices, and λ_*i*_ is an attenuation constant. Set the initial conditions of the system as $s\left(0\right)=0,\tilde{W}\left(0\right)=0,\tilde{c}\left(0\right)=0,ṽ\left(0\right)=0$; then Equation 29 can be re-expressed as:

(30) $$\begin{array}{l} \overset{m}{\underset{i=1}{\sum }}\int_{0}^{T}s_{i}^{2}\left(t\right)dt\leq \overset{m}{\underset{i=1}{\sum }}\lambda_{i}^{2}\int_{0}^{T}\omega_{i}^{2}\left(t\right)dt+\overset{m}{\underset{i=1}{\sum }}\int_{0}^{T}{\tilde{\nu }}_{i}^{2}\left(t\right)dt. \end{array}$$

To approximate an ideal sliding mode controller, assume that the approximation error between the proposed iFBEL and an ideal controller are bounded; in other words, ω ∈ *L*_2_[0, *T*_1_] and Ṽ ∈ *L*_2_[0, *T*_2_] with ∀*T*_1_, *T*_2_ ∈ [0, ∞]. Therefore $\int_{0}^{T}\omega_{i}^{2}\left(t\right)dt\leq N_{1}$ and $\int_{0}^{T}{\tilde{\nu }}_{i}^{2}\left(t\right)dt\leq N_{2}$, where *N*_1_ and *N*_2_ are two big positive constants. If λ = ∞, the minimum error cannot achieve approximation attenuation. If λ < ∞, the system is stable as shown by:

(31) $$\begin{array}{l} \overset{m}{\underset{i=1}{\sum }}\int_{0}^{T}s_{i}^{2}\left(t\right)dt\leq ||\lambda_{i}|{|}^{2}N_{1}+N_{2}<\infty . \end{array}$$

**Theorem 1**. *For the nonlinear system with Multiple Inputs and Multiple Outputs as represented by Equation 1, the proposed iFBEL can be described by Equation 24, in which the updating rule of the BEL is designed as expressed in Equation 32, and the adaptive laws of the FNN and robust controller are designed as stated in Equations (34-36)*.

(32) $$\begin{array}{ll} △V= & \;\alpha \left[\Gamma \times max\left(0,d-b\right)\right], \end{array}$$

(33) $$\begin{array}{ll} d= & \;\gamma \times p+\tau \times u_{BFC}, \end{array}$$

*where α is a learning-rate constant, and d consists of the input vector p and the output vector u_BFC_ with the learning constants γ and τ*.

(34) $$\begin{array}{l} \overset{\cdot }{Ŵ}=-\eta_{W}\hat{\Phi }s^{T}\left(\underline{e}\left(t\right)\right), \end{array}$$

(35) $$\begin{array}{l} \overset{\cdot }{ĉ}=-\eta_{c}\Phi_{c}^{T}Ŵs^{T}\left(\underline{e}\left(t\right)\right), \end{array}$$

(36) $$\begin{array}{l} \overset{\cdot }{\hat{v}}=-\eta_{v}\Phi_{v}^{T}Ŵs^{T}\left(\underline{e}\left(t\right)\right), \end{array}$$

(37) $$\begin{array}{l} u_{RC}={\left(2R^{2}\right)}^{-1}\left[\left(I+\Gamma^{2}\right)R^{2}+I\right]s^{T}\left(\underline{e}\left(t\right)\right), \end{array}$$

*where* $R=diag\left[\begin{array}{cccc} \lambda_{1} & \lambda_{2} & \ldots & \lambda_{m} \end{array}\right]\in {ℜ}^{m\times m}$ *is a diagonal matrix of a robust controller to converge the proposed system with the update rules* $\overset{\cdot }{Ŵ},\overset{\cdot }{ĉ}$ *and* $\overset{\cdot }{\hat{v}}$, *and* λ_*i*_ > 0, *where i = 1, 2, …, m; thus, R is a positive definite matrix*.

Proof. The Lyapunov function is given by:

(38) $$\begin{array}{l} V(s(\underline{e}(t)),\tilde{W},\tilde{V},\tilde{c},\tilde{v})=\frac{1}{2}[s^{T}(\underline{e}(t))G_{n}^{-1}s(\underline{e}(t))+tr[{\tilde{W}}^{T}\eta_{W}^{-1}\tilde{W}]\\ \;+{\tilde{c}}^{T}\eta_{c}^{-1}\tilde{c}+{\tilde{v}}^{T}\eta_{v}^{-1}\tilde{v}+tr[{\tilde{V}}^{T}\alpha^{-1}\tilde{V}]]. \end{array}$$

Taking the derivative of the Lyapunov function and using 28, yields

$$\begin{array}{ll} \;\overset{\cdot }{V}\left(s\left(\underline{e}\left(t\right)\right),\tilde{W},Ṽ,\tilde{c},ṽ\right)\\ = & s^{T}\left(\underline{e}\left(t\right)\right)G_{n}^{-1}ṡ\left(\underline{e}\left(t\right)\right)+tr\left[{\tilde{W}}^{T}\eta_{W}^{-1}\overset{\cdot }{\tilde{W}}\right]+{\tilde{c}}^{T}\eta_{c}^{-1}\overset{\cdot }{\tilde{c}}+{ṽ}^{T}\eta_{v}^{-1}\overset{\cdot }{ṽ}\\ +\;tr\left[{Ṽ}^{T}\alpha^{-1}\overset{\cdot }{Ṽ}\right]\\ = & s^{T}\left(\underline{e}\left(t\right)\right)G_{n}^{-1}ṡ\left(\underline{e}\left(t\right)\right)-tr\left[{\tilde{W}}^{T}\eta_{W}^{-1}\overset{\cdot }{Ŵ}\right]-{\tilde{c}}^{T}\eta_{c}^{-1}\overset{\cdot }{ĉ}-{ṽ}^{T}\eta_{v}^{-1}\overset{\cdot }{\hat{v}}\\ -tr\left[{Ṽ}^{T}\alpha^{-1}\overset{\cdot }{\hat{V}}\right]\\ = & s^{T}\left(\underline{e}\left(t\right)\right)Ṽ\hat{\Gamma }-s^{T}\left(\underline{e}\left(t\right)\right)\tilde{W}\hat{\Phi }-s^{T}\left(\underline{e}\left(t\right)\right)Ŵ\left(\Phi_{c}\tilde{c}+\Phi_{v}ṽ\right)\\ +\;s^{T}\left(\underline{e}\left(t\right)\right)\left(\omega -u_{RC}\right)-s^{T}\left(\underline{e}\left(t\right)\right)G_{n}^{-1}\sigma sgn\left[s\left(\underline{e}\left(t\right)\right)\right]\\ -tr\left[{\tilde{W}}^{T}\eta_{W}^{-1}\overset{\cdot }{Ŵ}\right]-{\tilde{c}}^{T}\eta_{c}^{-1}\overset{\cdot }{ĉ}-{ṽ}^{T}\eta_{v}^{-1}\overset{\cdot }{\hat{v}}-tr\left[{Ṽ}^{T}\alpha^{-1}\overset{\cdot }{\hat{V}}\right]\\ \leq -tr\left[\tilde{W}\left(s\left(\underline{e}\left(t\right)\right)\hat{\Phi }+\eta_{W}^{-1}\overset{\cdot }{Ŵ}\right)\right]-\tilde{c}\left[s^{T}\left(\underline{e}\left(t\right)\right)Ŵ\Phi_{c}+\eta_{c}^{-1}\overset{\cdot }{ĉ}\right]\\ -ṽ\left[s^{T}\left(\underline{e}\left(t\right)\right)Ŵ\Phi_{v}+\eta_{v}^{-1}\overset{\cdot }{\hat{v}}\right]+s^{T}\left(\underline{e}\left(t\right)\right)Ṽ\hat{\Gamma } \end{array}$$

(39) $$\begin{array}{l} +\;s^{T}\left(\underline{e}\left(t\right)\right)\left(\omega -u_{RC}\right). \end{array}$$

Since $\overset{\cdot }{\hat{V}}=0$ when *d*_*i*_ − *b* ≤ 0 and $\overset{\cdot }{\hat{V}}=\alpha \cdot \Gamma \cdot \left[d_{i}-b\right]>0$ if *d*_*i*_ − *a* > 0, consider Ṽ ∈ *L*_2_[0, *T*_2_] leading to $-tr\left[{Ṽ}^{T}\alpha^{-1}\overset{\cdot }{\hat{V}}\right]\leq 0$. Substituting 34–37 into 39, yields:

$$\begin{array}{ll} \;\overset{\cdot }{V}\left(s\left(\underline{e}\left(t\right)\right),\tilde{W},Ṽ,\tilde{c},ṽ\right)\\ \leq & s^{T}\left(\underline{e}\left(t\right)\right)Ṽ\hat{\Gamma }+s^{T}\left(\underline{e}\left(t\right)\right)\left(\omega -u_{RC}\right)\\ =s^{T}\left(\underline{e}\left(t\right)\right)Ṽ\hat{\Gamma }+s^{T}\left(\underline{e}\left(t\right)\right)\omega -\frac{1}{2}s^{T}\left(\underline{e}\left(t\right)\right)s\left(\underline{e}\left(t\right)\right)-\frac{1}{2}\frac{s^{T}\left(\underline{e}\left(t\right)\right)s\left(\underline{e}\left(t\right)\right)}{\lambda^{2}}\\ -\frac{1}{2}s^{T}\left(\underline{e}\left(t\right)\right)s\left(\underline{e}\left(t\right)\right)\hat{\Gamma }{\hat{\Gamma }}^{T}\\ =-\frac{1}{2}s^{T}\left(\underline{e}\left(t\right)\right)s\left(\underline{e}\left(t\right)\right)-\frac{1}{2}{\left[\frac{s\left(\underline{e}\left(t\right)\right)}{\lambda }-\lambda \omega \right]}^{2}-\frac{1}{2}{\left[s{\left(\underline{e}\left(t\right)\right)}^{T}\hat{\Gamma }-Ṽ\right]}^{2}\\ +\frac{1}{2}\lambda^{2}\omega^{2}+\frac{1}{2}{Ṽ}^{T}Ṽ \end{array}$$

(40) $$\begin{array}{ll} \leq & -\frac{1}{2}s^{T}\left(\underline{e}\left(t\right)\right)s\left(\underline{e}\left(t\right)\right)+\frac{1}{2}\lambda^{2}\omega^{2}+\frac{1}{2}{Ṽ}^{T}Ṽ. \end{array}$$

Integrating 40 from *t* = 0 to *t* = *T*, yields:

$$\begin{array}{l} V\left(T\right)-V\left(0\right)\leq -\frac{1}{2}\overset{m}{\underset{i=1}{\sum }}\int_{0}^{T}s_{i}^{2}\left(t\right)dt+\frac{1}{2}\overset{m}{\underset{i=1}{\sum }}\lambda_{i}^{2}\int_{0}^{T}\omega_{i}^{2}\left(t\right)dt \end{array}$$

(41) $$\begin{array}{l} +\frac{1}{2}\overset{m}{\underset{i=1}{\sum }}\int_{0}^{T}{\tilde{\nu }}_{i}^{2}\left(t\right)dt. \end{array}$$

Since *V*(*T*) > 0 and *V*(0) > 0, Equations 30 and 31 lead to $\overset{m}{\underset{i=1}{\sum }}\overset{T}{\underset{0}{\int }}s_{i}^{2}\left(t\right)dt<\infty$.

## 5. Experimentation

To verify the effectiveness and efficacy of the proposed controller with the new iFBEL, it was applied to two typical humanoid robotic systems, including a three-joint robot manipulator and a six-joint biped robot. A comparative study is also included in this section to evaluate the performance of the proposed controller in reference to two important control approaches including a PID controller and an SMC with fuzzy cerebellar model articulation controller network (FCMAC) (Lin et al., 2009).

PID control is a classic control method, which is linearly combined by proportional control, integral control and differential control. The FCMAC network has the characteristics of rapid convergence, which enable the work to be suitable for the robotic control. The effectiveness of the FCMAC-based network controller has been demonstrated in many recent studies, such as Lin et al. (2016) and Zhao and Lin (2017). The experiments of both three-joint robot manipulator and six-joint biped robot are simulated in MATLAB R2016a. The configuration of the algorithm computer is set as follows: The CPU and the operating system of the development computer are Intel Core i5-4200U CPU@2.30GHz and Windows 10 professional. The source code of the algorithm can be found in this link^1^.

The parameters for the robust controller and the iFBEL's Gaussian functions and weights are tuned by using Equations from 32 to 37. The learning rate parameters and iFBEL's network structure are set empirically.

### 5.1. Three-Joint Robot Manipulator

**Figure 4:**
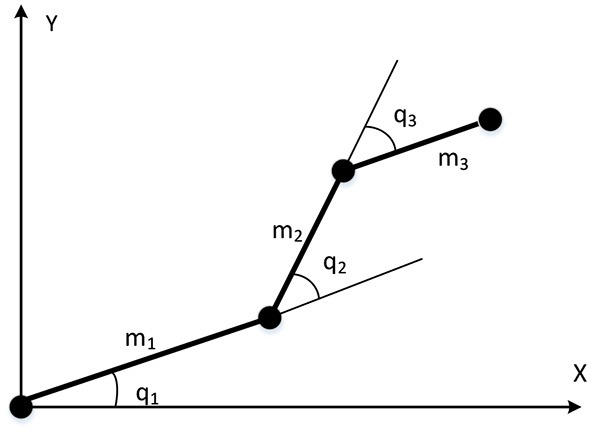
The three-links robot manipulator.

The first experiment was carried out using a relatively simple three-joint robot manipulator, to mainly practically evaluate the validity of the proposed system. The three-joint robot manipulator used in this experiment is illustrated in Figure 4; and the dynamic equation of such system is expressed as follows:

(42) $$\begin{array}{l} M\left(q\right)\ddot{q}+C\left(q,\overset{\cdot }{q}\right)\overset{\cdot }{q}+g\left(q\right)=u+\tau_{d}, \end{array}$$

where *q* ∈ ℜ^3^ is the joint angle state vector, $\overset{\cdot }{q}\in {ℜ}^{3}$ is the velocity vector, $\ddot{q}\in {ℜ}^{3}$ is the acceleration vector, *M*(*q*) ∈ ℜ^3×3^ is the inertia matrix, $C\left(q,\overset{\cdot }{q}\right)\in {ℜ}^{3\times 3}$ is the Coriolis/Centripetal matrix, *g*(*q*) ∈ ℜ^3^ is the gravity vector, and *q* = [−0.2, 0.5, −0.3]^*T*^, $\overset{\cdot }{q}=0$, $\ddot{q}=0$ are designated as the original state, *u* ∈ ℜ^3^ is the output torque. The detailed expression of $M\left(q\right),C\left(q,\overset{\cdot }{q}\right),g\left(q\right)$ and the nominal parameters of the manipulator are provided in Appendix 1.1.

The reference trajectories were given as $q_{d1}=\frac{1}{2}{\left[\frac{1}{2}(\sin(t+2.5)+0.7\cos(2t+1.5)],\sin(t)+\sin(2t),0.13-(\sin(t)+\sin(2t))\right]}^{T}$, $\overset{\cdot }{q_{d1}}=0$, $\ddot{q_{d1}}=0$. To evaluate the robustness of the proposed control system, the reference trajectories were modified as $q_{d2}=\frac{1}{2}{\left[\frac{1}{2}\left(\sin\left(2t\right)+\cos\left(t+1\right)\right),\sin\left(2t\right)+\cos\left(t+1\right),\cos\left(2t\right)-\sin\left(t\right)\right]}^{T}$, $\overset{\cdot }{q_{d2}}=0$, $\ddot{q_{d2}}=0$ at *t* = 15*s*, with the external disturbance of $\tau_{d}=\rho_{1}\times {\left[0.2\sin\left(2t\right),0.1\cos\left(2t\right),0.1\sin\left(t\right)\right]}^{T}$, where ρ_1_ = 1 is the amplification coefficient. In order to evaluate the proposed network's performances in various disturbance situations, two coefficients (ρ_1_ = 1.5 and ρ_1_ = 2) were also used in the experiments. The BEL and the FNN were characterized as follows:

- the number of elements for each state variable: *n*_*E*_ = 5 (elements);
- generalization: *n*_*C*_ = 4 (elements/block);
- the number of blocks for each state variable for both the BEL and FNN: *n*_*b*_ = *n*_*f*_ = 2 (blocks/layer) × 4 (layer) = 8 (blocks);
- the number of receptive fields: *n*_*E*_ = 2 (receptive fields/layer) × 4 (layer) = 8 (receptive fields).

The initial means of the Gaussian functions in the Association Memory Spaces were divided equally and set as [−1, 1] for the BEL, and [−2, 2] for the FNN. The initial variances were set as σ_*ij*_ = 0.1 for the BEL, and σ_*pq*_ = 0.1 for the FNN, where *i* = *p* = 1, 2, 3, and *j* = *q* = 1, 2…, 8. The weights of both the BEL and the FNN were initialized as zero and then automatically adjusted during the online training process. In addition, the learning rates were set as follows: η_ω_ = 20, η_*m*_ = 0.001, η_*v*_ = 0.001, α = 0.01, *b* = 0.1, *c* = 0.1.

The parameters of PID controller in the comparison experiments were set as: κ_*P*_ = 15, κ_*I*_ = 0.2, κ_*D*_ = 0.5, where κ_*P*_, κ_*I*_ and κ_*D*_ are the coefficients of the proportional controller, integral controller and differential controller. FCMAC controller in the comparison experiments has the same parameters as FNN does.

**Figure 5:**
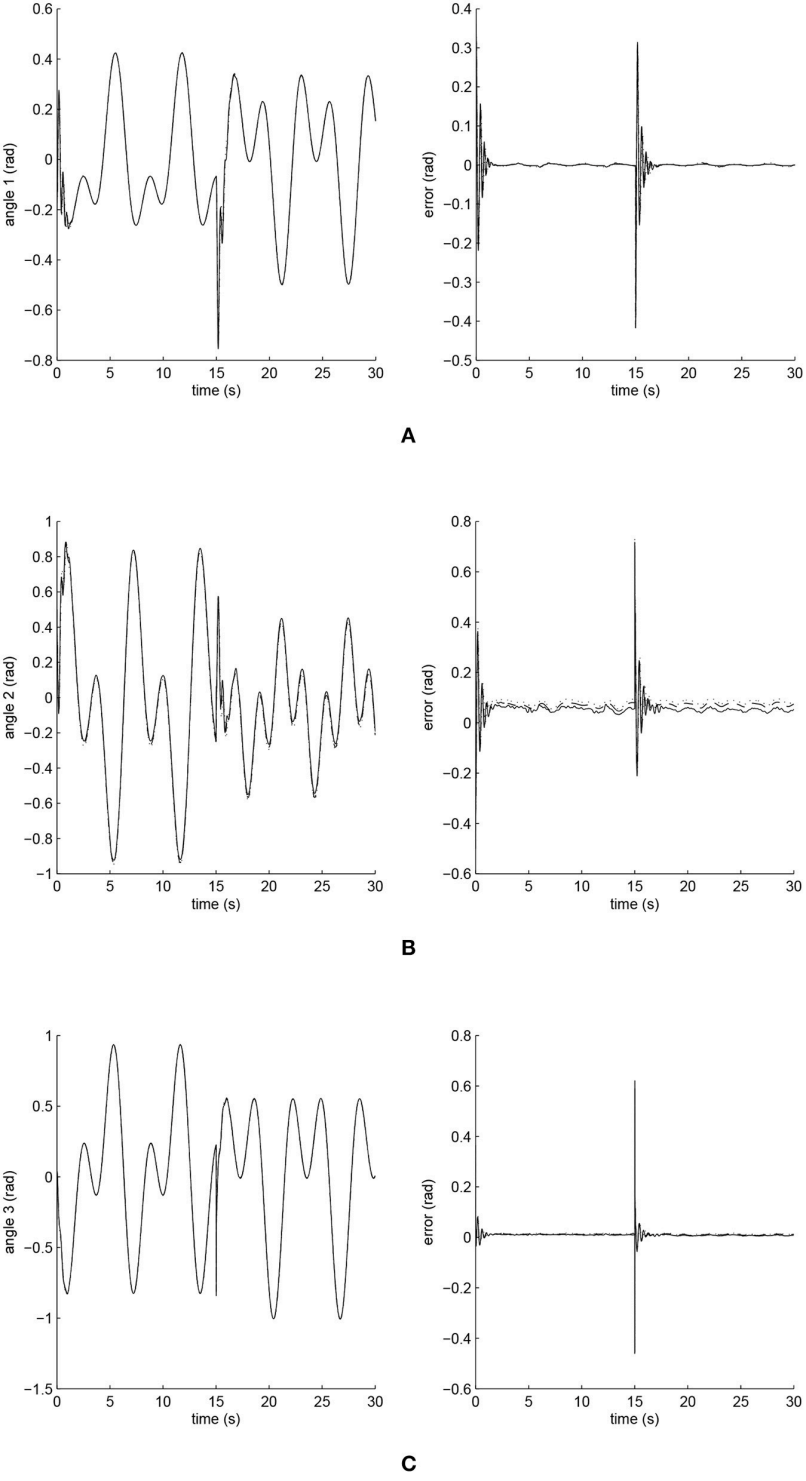
Trajectory responses (in the left) and tracking errors (in the right) of Joints 1, 2, and 3 at ρ_1_ = 1. The solid line indicates the performance of the iFBEL; the dotted line represents that of the PID controller; and the dot dash line implies that of the FCMAC controller. **(A)** Trajectory response and tracking error of Joint 1. **(B)** Trajectory response and tracking error of Joint 2. **(C)** Trajectory response and tracking error of Joint 3.

**Figure 6:**
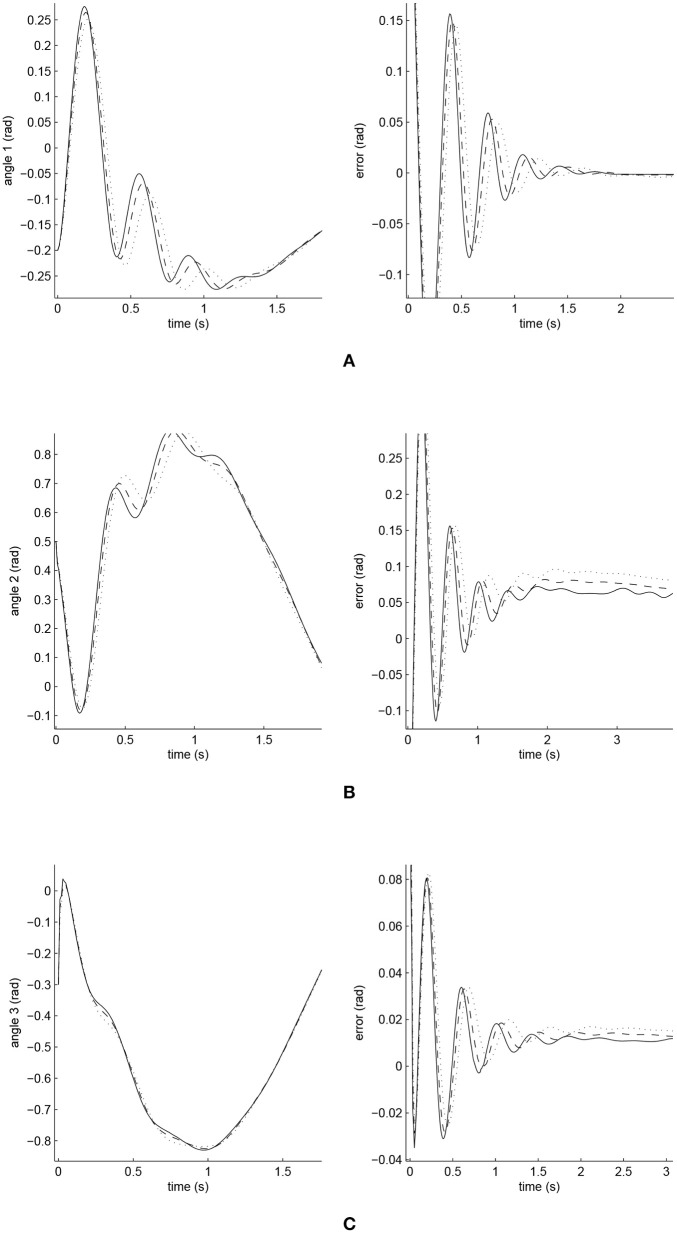
Amplified trajectory responses (in the left) and tracking errors (in the right) at *t* = 0 of Joints 1, 2, and 3. The solid line indicates the performance of the iFBEL; the dotted line represents that of the PID controller; and the dot dash line implies that of the FCMAC controller. **(A)** Amplified trajectory response and tracking error of Joint 1. **(B)** Amplified trajectory response and tracking error of Joint 2. **(C)** Amplified trajectory response and tracking error of Joint 3.

**Figure 7:**
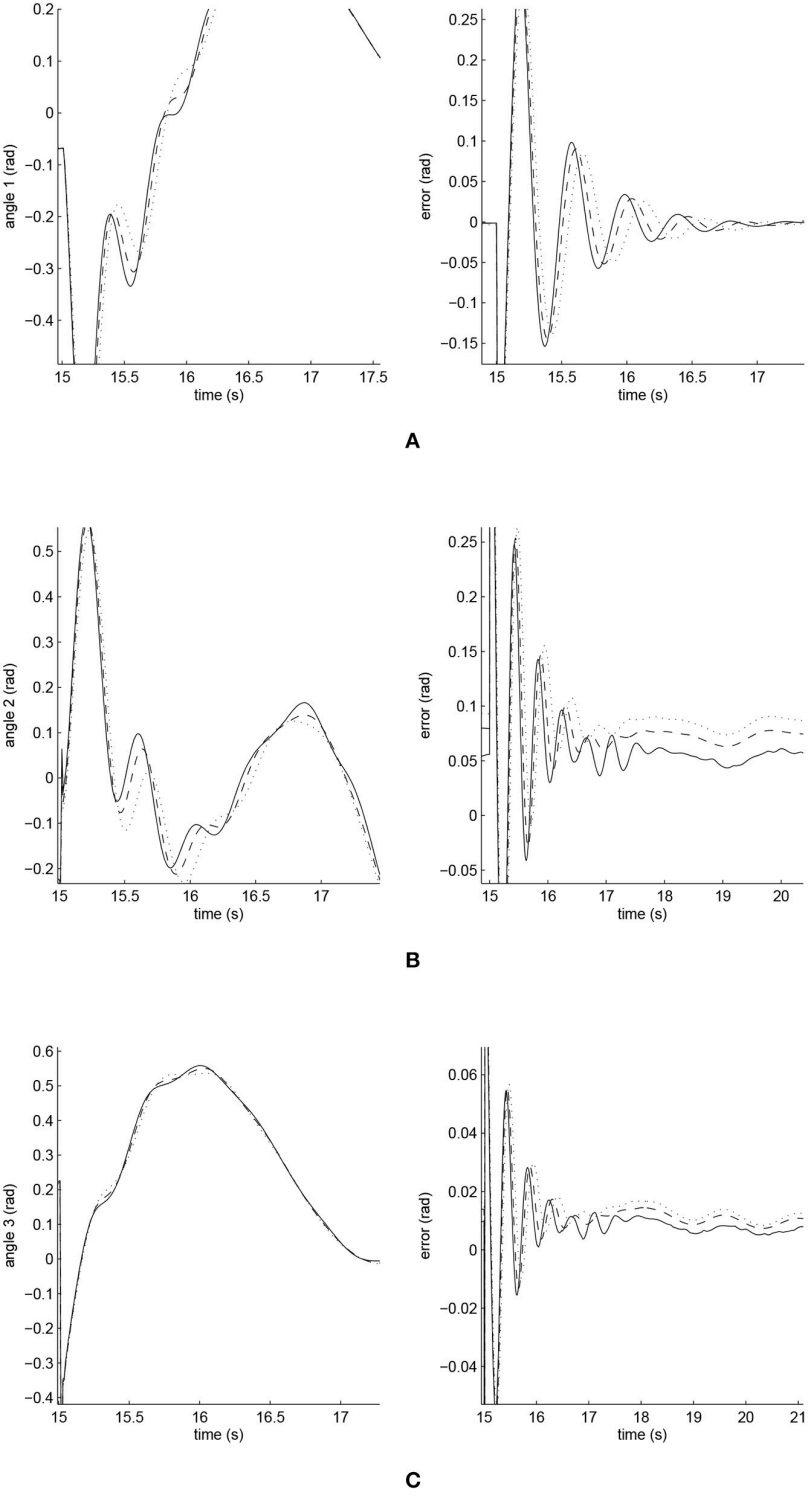
Amplified trajectory responses (in the left) and tracking errors (in the right) at *t* = 15 of Joints 1, 2, and 3. The solid line indicates the performance of the iFBEL; the dotted line represents that of the PID controller; and the dot dash line implies that of the FCMAC controller. **(A)** Amplified trajectory response and tracking error of Joint 1. **(B)** Amplified trajectory response and tracking error of Joint 2. **(C)** Amplified trajectory response and tracking error of Joint 3.

The simulated position responses and the tracking errors at ρ_1_ = 1 are shown in Figure 5. To better distinguish these values for the three controllers, Figures 6, 7 show the amplified trajectory responses and the tracking errors at *t* = 0 and *t* = 15. In Figure 6, the PID controller required 1.4*s*, 1.3*s*, and 0.05*s* for Joints 1, 2, and 3 to converge, respectively, while the FCMAC required 1.2*s*, 1.3*s*, and 0.05*s* for these joints respectively; however, the proposed iFBEL controller just needed 1.1*s*, 1.3*s*, 0.03*s* for these joints, respectively. In addition, the iFBEL performed the best when *t* = 15*s*.

The accumulated RMSE values at ρ_1_ = 1 during the entire experiment are listed in Table 1, which also proved that the proposed iFBEL controller outperformed others. However, the difference among the three controllers is insignificant. The FCMAC and the PID controllers also generated good control performances in this experiment, because the three-joint manipulator system is not very complicated. The accumulated RMSE values under ρ_1_ = 1.5 and ρ_1_ = 2 are listed in Tables 2, 3, respectively. With the increase of disturbance, the errors of the three controllers also increased. However, the iFBEL also achieved the best performance under the two disturbance situations. This proves that the proposed iFBEL can well handle larger disturbances.

**Table 1:** The accumulated RMSE values of each joint at ρ_1_ = 1.

	**PID**	**FCMAC**	**iFBEL**
Joint 1	3.434986*e*−002	3.351934*e*−002	3.349444*e*−002
Joint 2	9.348720*e*−002	8.239905*e*−002	6.913146*e*−002
Joint 3	2.224425*e*−002	2.143444*e*−002	2.027774*e*−002

**Table 2:** The accumulated RMSE values of each joint at ρ_1_ = 1.5.

	**PID**	**FCMAC**	**iFBEL**
Joint 1	3.682201*e*−002	3.359658*e*−002	3.352264*e*−002
Joint 2	1.007825*e*−001	8.268712*e*−002	6.936621*e*−002
Joint 3	2.361193*e*−002	2.148857*e*−002	2.030388*e*−002

**Table 3:** The accumulated RMSE values of each joint at ρ_1_ = 2.0.

	**PID**	**FCMAC**	**iFBEL**
Joint 1	4.266091*e*−002	3.370990*e*−002	3.359901*e*−002
Joint 2	2.177750*e*−001	8.390025*e*−002	7.112548*e*−002
Joint 3	2.695101*e*−002	2.155297*e*−002	2.034952*e*−002

### 5.2. The Biped Robot

**Figure 8:**
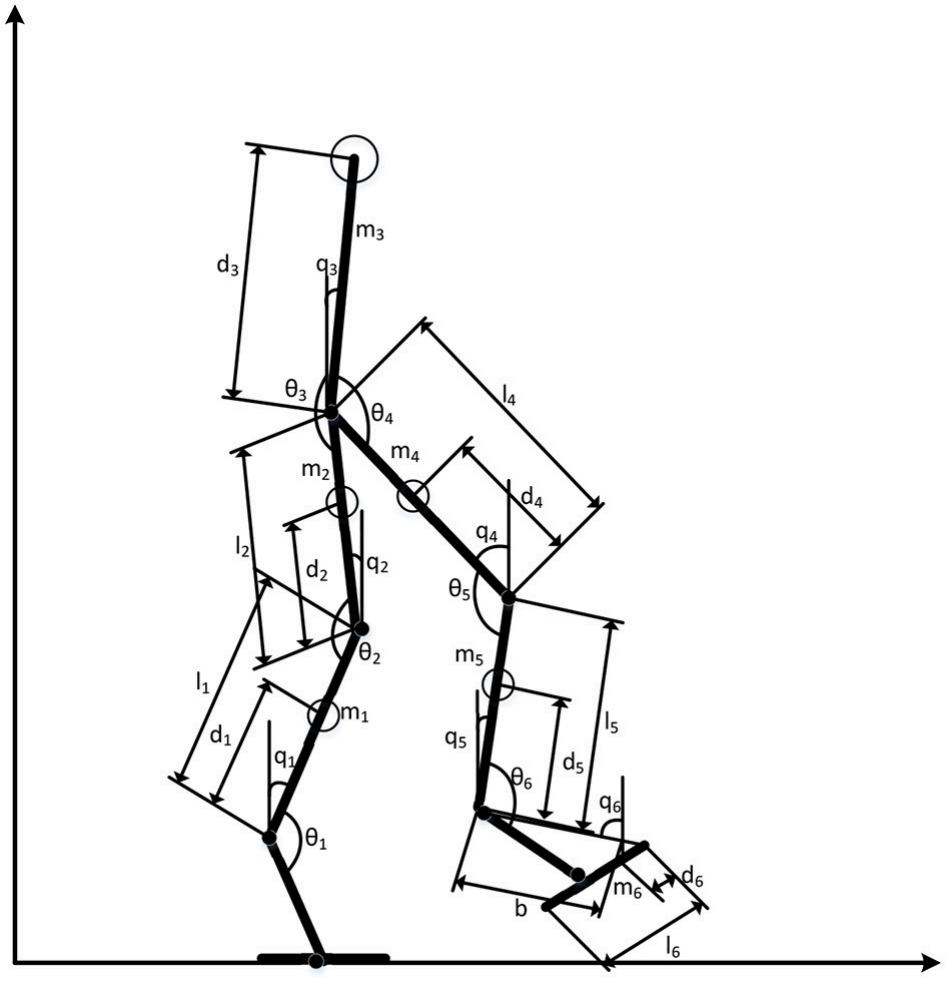
The six-links biped robot used in the experiment.

The configuration of the six-link biped robot used in this second experiment is illustrated in Figure 8. The experiment reported in the last sub-section was mainly used to validate the proposed system, but the experiment reported in this sub-section was primarily used to evaluate the efficiency and efficacy of the proposed control system. The dynamic equation of the robot is given as follow:

(43) $$\begin{array}{l} M\left(q\right)\ddot{q}+C\left(q,\overset{\cdot }{q}\right)\overset{\cdot }{q}+g\left(q\right)=u+\tau_{d}, \end{array}$$

where *q* ∈ ℜ^6^, $\overset{\cdot }{q}\in {ℜ}^{6}$, $\ddot{q}\in {ℜ}^{6}$ are the joint angle state vector, velocity vector and acceleration vector respectively, and *M*(*q*) ∈ ℜ^6×6^, $C\left(q,\overset{\cdot }{q}\right)\in {ℜ}^{6\times 6}$, *g*(*q*) ∈ ℜ^6^ are the inertia matrix, the Coriolis/Centripetal matrix and the gravity vector respectively, *u* ∈ ℜ^6^ is the output torque. More details for $M\left(q\right),C\left(q,\overset{\cdot }{q}\right),g\left(q\right)$ and the nominal parameters of the biped robot can be found in Appendix 1.2.

This experiment also considered the phases of signal support of a gait cycle. The analysis planning and walking pattern generation are detailed in Appendix 1.3. The generated gait trajectory $q_{d}={\left[\theta_{1},\theta_{2},\ldots ,\theta_{6}\right]}^{T}$,$\overset{\cdot }{q_{d}}=0$, $\ddot{q_{d}}=0$ were set as the reference trajectories of the biped robot. The initial angles of each joint were given as *q* = [0.37, 0.5, 0.75, −0.15, −0.56, 0.85]^*t*^,$\overset{\cdot }{q}=0$, $\ddot{q}=0$. τ_*d*_ = ρ_2_ × *exp*(−0.1*t*)_6×1_ was used in this experiment as the external disturbance, where ρ_2_ = 1 is the amplification coefficient.

The BEL and the FNN are characterized as the same with that used in the first experiment as reported in section 5.1, but with different initializations. In particular, the initial means of the Gaussian functions in the Association Memory Spaces in this experiment were divided equally and set as [−1.4, 1.4] for the BEL, and [−1.6, 1.6] for the FNN. The initial variances were set as σ_*ij*_ = 0.01 for the BEL and σ_*pq*_ = 0.5 for the FNN, where *i* = *p* = 1, 2…, 6, and *j* = *q* = 1, 2…, 8. The weights of both sub-systems were initialized from zero and then automatically adjusted during the online training stage. In this experiment, the learning rates were chosen as η_ω_ = 0.01, η_*m*_ = 0.001, η_*v*_ = 0.001, α = 0.01, *b* = 0.05, and *c* = 0.01.

The parameters of PID controller in the second experiment were set as: κ_*P*_ = 8, κ_*I*_ = 0.5, κ_*D*_ = 1.3. FCMAC controller in the second experiment also has the same parameters as FNN does.

**Figure 9:**
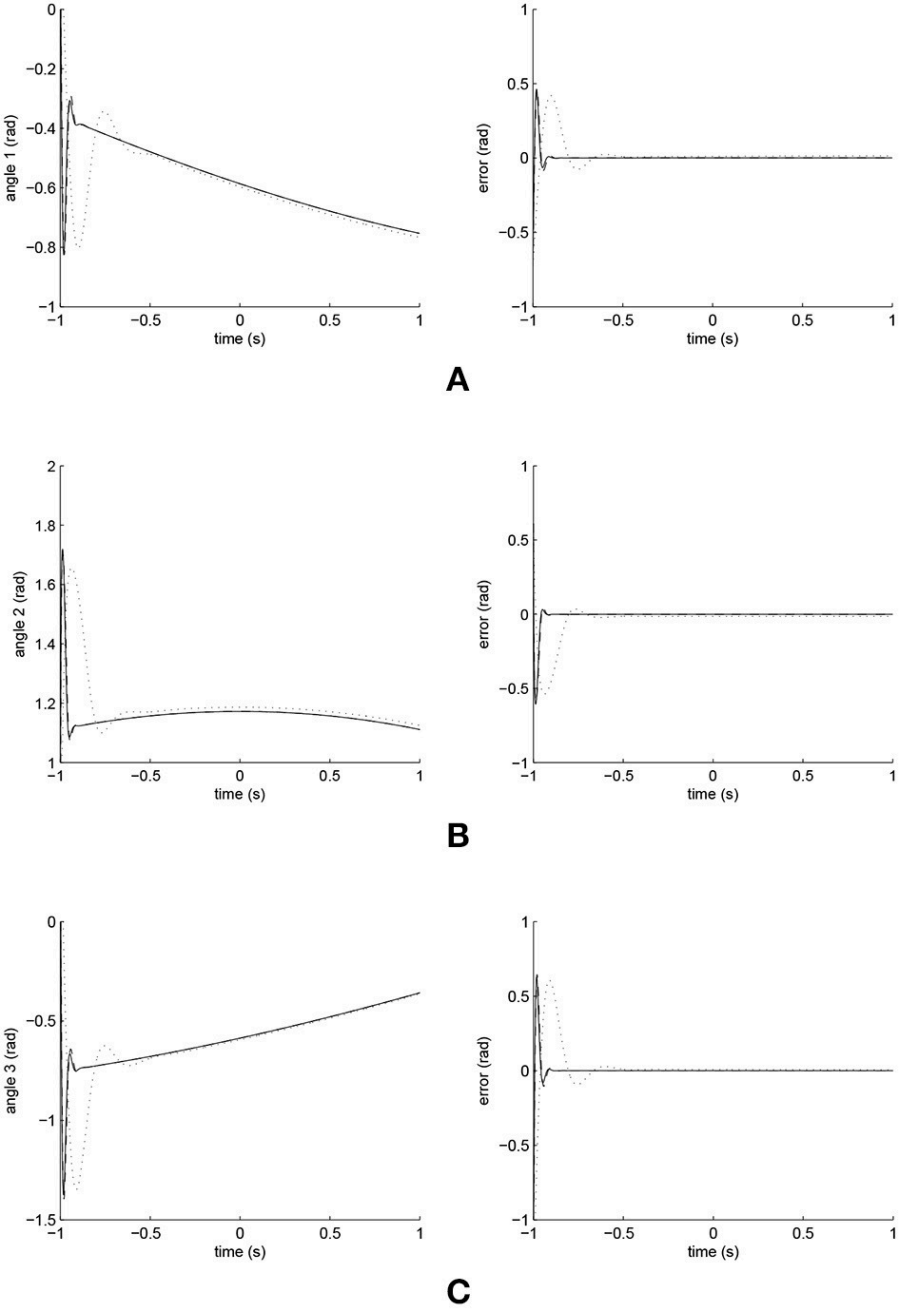
Trajectory responses (in the left) and tracking errors (in the right) of Joints 1, 2, and 3 at ρ_2_ = 1. The solid line indicates the performance of the iFBEL; the dotted line represents that of the PID controller; and the dot dash line implies that of the FCMAC controller. **(A)** Trajectory response and tracking error of Joint 1. **(B)** Trajectory response and tracking error of Joint 2. **(C)** Trajectory response and tracking error of Joint 3.

**Figure 10:**
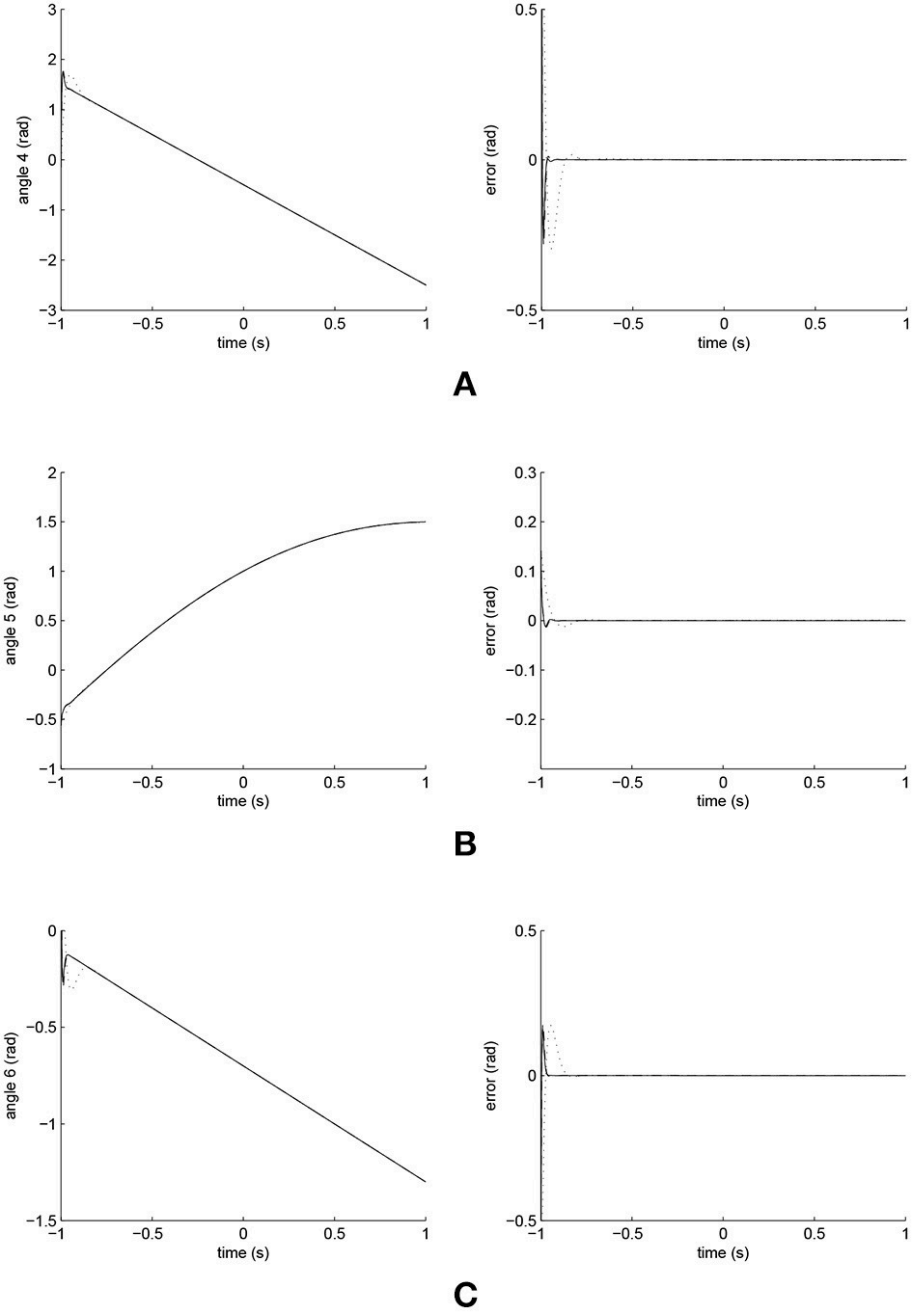
Trajectory responses (in the left) and tracking errors (in the right) of Joints 4, 5, and 6 at ρ_2_ = 1. The solid line indicates the performance of the iFBEL; the dotted line represents that of the PID controller; and the dot dash line implies that of the FCMAC controller. **(A)** Trajectory response and tracking error of Joint 4. **(B)** Trajectory response and tracking error of Joint 5. **(C)** Trajectory response and tracking error of Joint 6.

**Figure 11:**
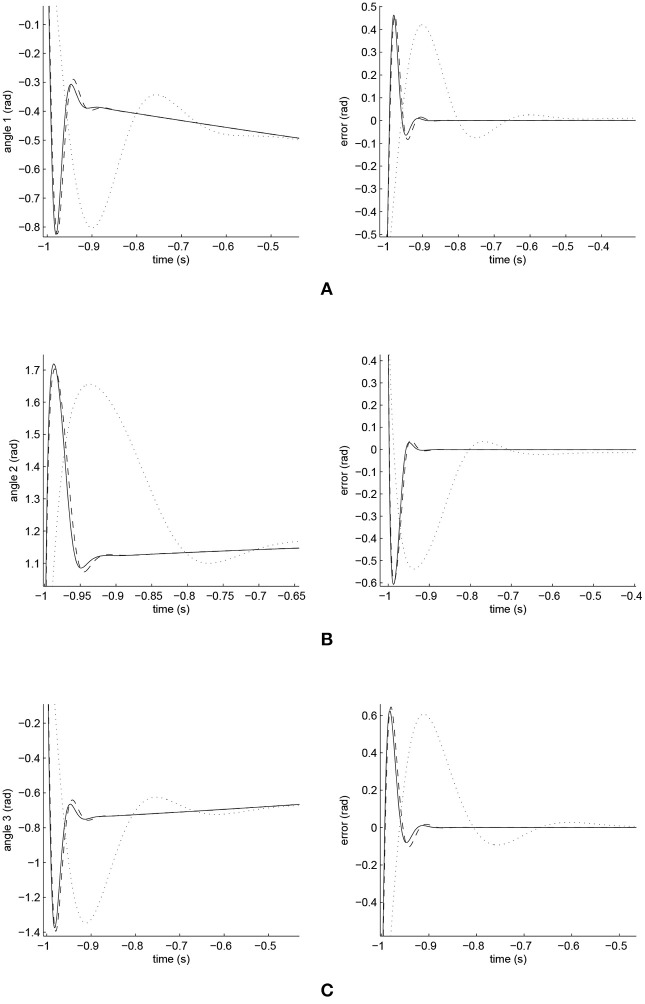
Amplified trajectory responses (in the left) and tracking errors (in the right) of Joints 1, 2, and 3. The solid line indicates the performance of the iFBEL; the dotted line represents that of the PID controller; and the dot dash line implies that of the FCMAC controller. **(A)** Amplified trajectory response and tracking error of Joint 1. **(B)** Amplified trajectory response and tracking error of Joint 2. **(C)** Amplified trajectory response and tracking error of Joint 3.

**Figure 12:**
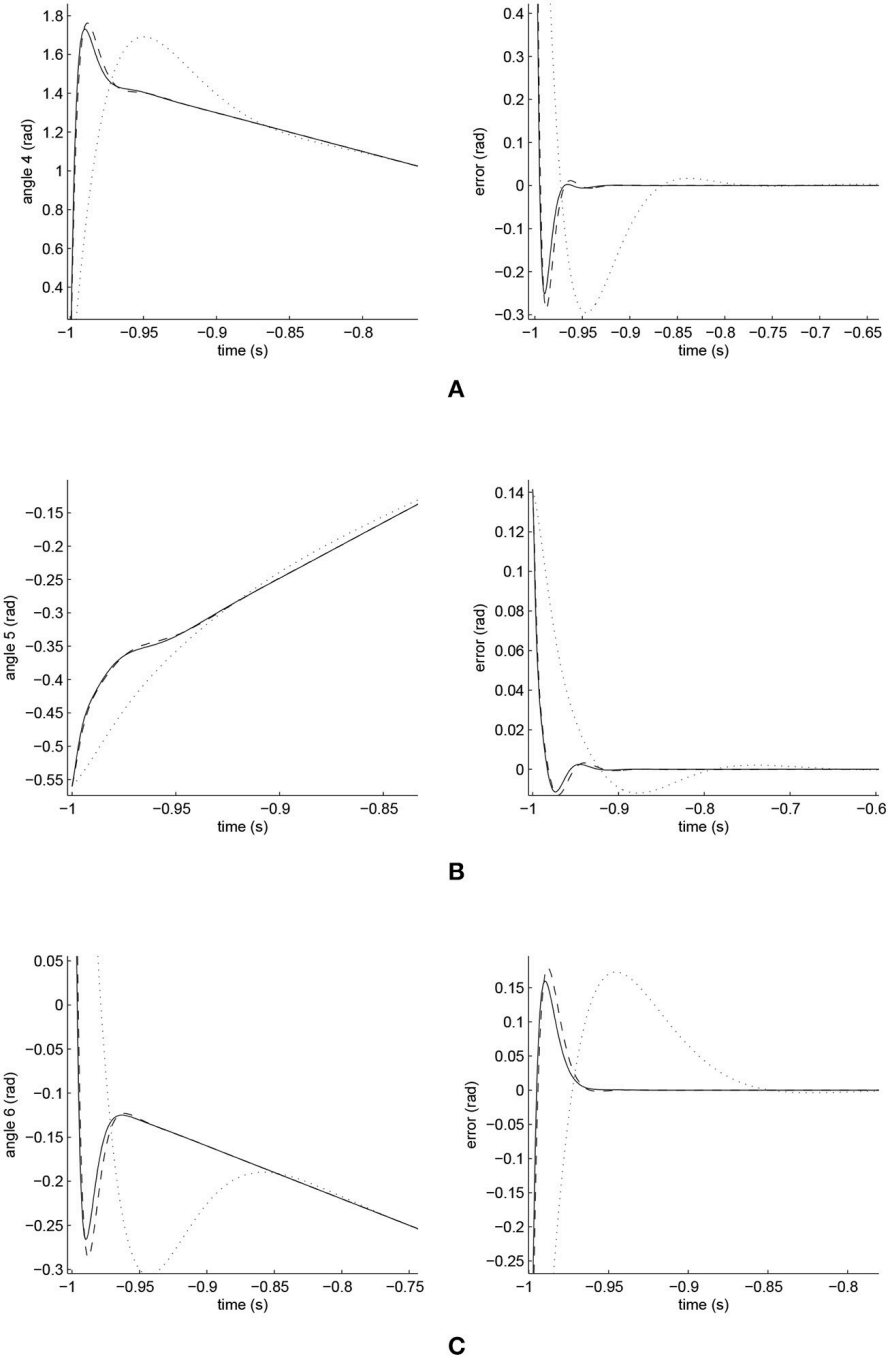
Amplified Trajectory responses (in the left) and tracking errors (in the right) of Joints 4, 5, and 6. Solid line indicate iFBEL with the dotted one point PID controller and the dot dash one imply FCMAC controller. **(A)** Amplified trajectory response and tracking error of Joint 4. **(B)** Amplified trajectory response and tracking error of Joint 5. **(C)** Amplified trajectory response and tracking error of Joint 6.

The simulated position responses and the tracking errors at ρ_2_ = 1 led by the three controllers are illustrated in Figures 9, 10; with the performances of Joints 1, 2 and 3 illustrated in Figure 9 and those of Joints 4, 5, and 6 in Figure 10. The PID controller had a significant convergence delay, which therefore represented the worst performance within the three controllers. It is difficult from these figures to distinguish the performances led by the FCMAC and the iFBEL controllers, and thus the trajectories resulted from all the controllers in the range of [−1.4*s*, 1.4*s*] are magnified as displayed in Figures 11, 12 for better visualization and thus easier investigation.

From Figures 11, 12, it is clear that the PID controller could not converge rapidly in all the joints of the biped robot. The performances of the FCMAC and the iFBEL regarding all of the joints were very similar; both controllers rapidly converged the tracking errors. The tracking error amplitudes of the FCMAC controller in Joints 1, 2, 3, and 6 were larger than those of the iFBEL controller, which indicates the superiority of the proposed iFBEL controller.

The accumulated RMSE values are listed in Table 4. It is clear from this table that the convergence time of the iFBEL controller was shorter than those of the PID and the FCMAC for each joint. In this case, the RMSE values also proved that the proposed iFBEL controller achieved the best control performance within the three compared controllers used in this comparative study. The accumulated RMSE values at ρ_2_ = 1.5 and ρ_2_ = 2 are also given in Tables 5, 6, respectively. The iFBEL also achieved the best performance under the two disturbance situations.

**Table 4:** The accumulated RMSE value of each joint of the biped robot at ρ_2_ = 1.

	**PID**	**FCMAC**	**iFBEL**
Joint 1	1.425845*e*−001	7.393872*e*−002	7.000116*e*−002
Joint 2	1.629218*e*−001	8.733478*e*−002	8.542263*e*−002
Joint 3	2.259217*e*−001	1.157991*e*−001	1.074686*e*−001
Joint 4	1.510193*e*−001	7.791578*e*−002	7.197584*e*−002
Joint 5	1.942511*e*−002	9.005906*e*−003	8.473318*e*−003
Joint 6	8.756004*e*−002	4.523769*e*−002	4.179952*e*−002

**Table 5:** The accumulated RMSE value of each joint of the biped robot at ρ_2_ = 1.5.

	**PID**	**FCMAC**	**iFBEL**
Joint 1	2.263701*e*−001	7.555302*e*−002	7.184853*e*−002
Joint 2	1.780980*e*−001	8.710034*e*−002	8.501013*e*−002
Joint 3	2.873682*e*−001	1.158061*e*−001	1.087909*e*−001
Joint 4	1.990493*e*−001	8.022518*e*−002	7.258786*e*−002
Joint 5	1.420940*e*−001	3.344871*e*−002	1.409585*e*−002
Joint 6	1.699252*e*−001	4.900036*e*−002	4.357163*e*−002

**Table 6:** The accumulated RMSE value of each joint of the biped robot at ρ_2_ = 2.

	**PID**	**FCMAC**	**iFBEL**
Joint 1	4.839124*e*−001	8.177531*e*−002	7.763216*e*−002
Joint 2	4.010141*e*−001	8.750089*e*−002	8.712830*e*−002
Joint 3	5.154115*e*−001	1.158737*e*−001	1.128520*e*−001
Joint 4	4.370271*e*−001	8.295892*e*−002	7.663251*e*−002
Joint 5	4.213654*e*−001	5.440431*e*−002	2.932427*e*−002
Joint 6	4.350418*e*−001	5.853378*e*−002	5.095499*e*−002

## 6. Discussion

A humanoid robot usually consists of multiple joints and suffers many unexpected disturbances; therefore, the controller of humanoid robot must own the powerful non-linear approximation ability to handle these complex situations. Based on the results of the two simulations, the proposed iFBEL network successfully demonstrated a rapid convergence ability and a nonlinear mapping capability. In the two simulations, the iFBEL controller can always achieve the fastest reaction speed to reduce errors; in addition, the iFBEL controller still achieved the best performance in different disturbance patterns. Therefore, the proposed network is suitable for the control of humanoid robots.

Although the performance of iFBEL-based controller was better than those of the FCMAC and PID controllers, the iFBEL network's structure is more complicated than that of the FCMAC. To address this issue, we believe that a recurrent mechanism usually uses a simple network structure to achieve good dynamic performance. Therefore, in the future work, we will improve our method by embedding a recurrent network inside the iFBELC controller.

## 7. Conclusion

This paper proposed a novel humanoid robot controller, which integrates some components from a fuzzy neural network and a brain emotional learning model into a sliding mode controller for dynamic non-linear control. It has been theoretically proven that the proposed system is asymptotically stable, thus guaranteeing the convergence. Experimental results and comparative studies further verified this, and demonstrated precise position tracking, more favorable stability, and better performance in reference to the results generated from the recently-developed network controllers of PID and FCMAC.

This research can be further improved in several directions. The current iFBEL network does not include any recurrent mechanism, but such a mechanism can generally improve the dynamic performance of a network. Therefore, a future investigation will focus on the development of the recurrent feature to better support the iFBEL controller. In addition, the undesired chattering situation existing in the sliding surface has not been fully investigated; more efforts will focus on this issue. Furthermore, the proposed approach was only practically applied to the dynamic humanoid robot control in this work. It is worthwhile to apply the approach to a wider range of applications to fully discover its potential.

## Author Contributions

WF contributed to this work by developing the proposed method and preparing the experiments. FC contributed the implementation of the proposed method and writing the manuscript. C-ML conducted the statistical analysis of the experimental results. LY contributed the planning and analysis of the experiments and writing of the manuscript. CS contributed to the design of the proposed method. CZ contributed to the writing of the manuscript.

### Conflict of Interest Statement

The authors declare that the research was conducted in the absence of any commercial or financial relationships that could be construed as a potential conflict of interest.

## Footnotes

^1^https://github.com/Xiaomu-Shan/Experiments-of-iFBEL

## Abbreviations

BEL: Brain Emotional Learning
BFC: BEL and FNN Controller
FCMAC: Fuzzy Cerebellar Model Articulation Controller
FNN: Fuzzy Neural Network
iFBEL: improved Fuzzy Brain Emotional Learning
ISMC: Ideal Sliding Model Controller
PID: Proportional Integral Derivative
RC: Robust Controller
RMSE: Root Mean Square Error
SMC: Sliding Mode Control.
