# Supplementary material for: An Improved Fuzzy Brain Emotional Learning Model Network Controller for Humanoid Robots
Source: Front Neurorobot. 2019 Feb 4;13:2. doi: 10.3389/fnbot.2019.00002 (PMC6369368; doi:10.3389/fnbot.2019.00002)
Supplement: Supplementary file 1 [file Appendix.pdf]

1 APPENDIX

1.1 The nominal parameters of robot manipulator

The detailed description of the dynamic equation of the three-links robot manipulator are given as follows:

$$M(q) = \{bq_{ij}\} \in \Re^{3\times 3}$$

$$C(q,\dot{q}) = \{\sum_{k=1}^{3} cq_{ijk}q_k\} \in \Re^{3\times 3}$$

$$g(q) = \{gq_i\} \in \Re^{3}$$

for $i, j = 1, 2, 3$, we have:

$$\begin{aligned} bq_{11} =& I_1 + I_2 + I_3 + 0.15\cos^2(q_2) \\ &+ m_2 l_2^2 \cos^2(q_2) + m_3(0.5\cos(q_2) \\ &+ l_3\cos(q_2+q_3))^2 + 0.036 \end{aligned}$$

$$bq_{12} = bq_{13} = bq_{21} = bq_{31} = 0$$

$$\begin{aligned} bq_{22} =& I_2 + I_3 + m_2 l_2^2 + \\ & m_3(l_3^2 + l_3\cos(q_2) + 0.25) + 0.174 \end{aligned}$$

$$\begin{aligned} bq_{23} =& bq_{32} = I_3 + m_3 l_3^2 + 0.5 m_3 l_3 \cos(q_3) \\ &+ 0.012 \end{aligned}$$

$$bq_{33} = I_3 + m_3 l_3^2 + 0.012, cq_{112} = cq_{121}$$

$$\begin{aligned} cq_{111} &= cq_{122} = cq_{123} = cq_{133} = cq_{222} \\ &= cq_{313} = cq_{323} = cq_{331} = cq_{332} \\ &= cq_{333} = 0 \end{aligned}$$

$$\begin{aligned} cq_{112} =& -m_3(0.5\cos(q_2) + l_3\cos(q_2+q_3)) \\ &\times (0.5\sin(q_2) + l_3\sin(q_2+q_3)) \\ &- (0.15 + m_2 l_2^2)\sin(q_2) \end{aligned}$$

$$\begin{aligned} cq_{113} =& cq_{131} = -m_3(0.5\cos(q_2) \\ &+ l_3\cos(q_2+q_3))l_3\sin(q_2+q_3) \end{aligned}$$

$$\begin{aligned} cq_{132} =& cq_{123}, cq_{211} = -cq_{112}, cq_{212} = -cq_{122}, \\ & cq_{213} = -cq_{123}, cq_{221} = -cq_{122} \end{aligned}$$

$$cq_{223} = cq_{232} = cq_{233} = -0.5 m_3 l_3 \sin(q_3)$$

$$\begin{aligned} cq_{311} =& -cq_{113}, cq_{312} = cq_{213}, cq_{321} = cq_{312}, \\ & cq_{322} = -cq_{223}, cq_{231} = cq_{213} \end{aligned}$$

$$gq_1 = 0, gq_3 = l_3 m_3 g\cos(q_2 + q3)$$

$$\begin{aligned} gq_2 =& (0.5\cos(q_2) + l_3\cos(q_2+q_3))m_3 g \\ &+ l_2 m_2 g\cos(q_2) + 0.3g\cos(q_2). \end{aligned}$$

The link parameters of this robot manipulator are provided in Table 1.

1.2 The nominal parameters of biped robot

The biped robot demonstrated in Fig.6 has six links whose left leg is the supporting leg with Joint 1 and 2 and the right leg is the swing leg with Joint 6 and 5. The detailed description of the dynamic equation is given as follows:

$$M(q) = \{\delta_{ij}\cos(q_i - q_j)\} \in \Re^{6\times 6}$$

$$C(q,\dot{q}) = \{\delta_{ij}\sin(q_i - q_j)\} \in \Re^{6\times 6}$$

$$g(q) = \{h_i \sin(q_i)\} \in \Re^{6},$$

for $i, j = 1, 2, \ldots, 6$, we have:

$$\begin{aligned} \delta_{11} =& (m_2 + m_3 + m_4 + m_5 + m_6) l_1^2 \\ & m_1 d_1^2 + + I_1 \end{aligned}$$

$$\delta_{22} = m_2 d_2^2 + (m_3 + m_4 + m_5 + m_6) l_2^2 + I_2$$

$$\delta_{33} = m_3 d_3^2 + I_3$$

$$\delta_{44} = m_4(l_4 - d_4)^2 + (m_5 + m_6) d_4^2 + I_4$$

$$\delta_{55} = m_5(l_5 - d_5)^2 + m_6 l_5^2 + I_5$$

$$\delta_{66} = m_6 b^2 + I_5$$

$$\delta_{12} = m_2 l_1 d_2 + (m_3 + m_4 + m_5 + m_6) l_1 l_2$$

$$\delta_{13} = m_3 l_1 d_3$$

$$\delta_{14} = -m_4 l_1(l_4 - d_4) - (m_5 + m_6) l_1 l_4$$

$$\delta_{15} = -m_5 l_1(l_5 - d_5) - m_6 l_1 l_5$$

$$\delta_{16} = -m_6 l_1 b, \delta_{23} = m_3 l_2 d_3$$

$$\delta_{24} = -m_4 l_2(l_4 - d_4) - (m_5 + m_6) l_2 l_4$$

$$\delta_{25} = -m_5 l_2(l_5 - d_5) - m_6 l_2 l_5$$

$$\delta_{26} = -m_6 l_2 b, \delta_{34} = \delta_{35} = \delta_{36} = 0$$

$$\delta_{45} = m_5 l_4 (l_5 - d_5) + m_6 l_4 l_5, \delta_{46} = m_6 l_4 b$$

$$\delta_{56} = m_6 l_5 b$$

$$\delta_{ij} = \delta_{ji} \text{ , for } i = 1, 2, \ldots, 6 \text{ and } j = 1, 2, \ldots, 6$$

$$h_1 = (m_1 d_1 + m_2 l_1 + m_3 l_1 + m_4 l_1 + m_5 l_1 + m_6 l_1) g$$

$$h_2 = (m_2 d_2 + m_3 l_2 + m_4 l_2 + m_5 l_2 + m_6 l_2) g$$

$$h_3 = m_3 d_3 g$$

$$h_4 = (m_4 d_4 - m_4 l_4 - m_5 l_4 - m_6 l_4) g$$

$$h_5 = (m_5 d_5 - m_5 l_5 - m_6 l_5) g, h_6 = -m_6 b g.$$

In addition, Table 2 provides the link parameters of this biped robot.

1.3 Gait pattern

One cycle of robot walking is composed of a double support phase and a single support phase. This experiment considers just the phase of single support characterized as one foot swing in the air and another foot supporting on the ground. There are two ways to generate a walking pattern for a biped robot Huang et al. (2002). Observing a walking human and then planning the gait of a robot is a simple, but indeterminate, way because it is less confident in the physical stability. If the Zero Moment Point (ZMP) is within the convex hull of the supporting foot, the biped robot will be stable in her walk. Planning the curve of the ZMP and obtaining the path of the ankle and the hip, the trajectory of each joint of the two legs is derived by the kinematics and dynamics, which was the second approach used in this paper. Let the height of the hip be a constant $H = 0.5m$ and the trajectory of the hip in X direction be $T(t) = 0.1t$. From the kinematic relation Shih et al. (1990), the trajectory of each joint can be represented by:

$m_1 = 3kg$	$l_1 = 0.3m$	$I_1 = 50.45 \times 10^{-3} kgm^2$
$m_2 = 1.8kg$	$l_2 = 0.25m$	$I_2 = 32.68 \times 10^{-3} kgm^2$
$m_3 = 1.5kg$	$l_3 = 0.2m$	$I_3 = 30.47 \times 10^{-3} kgm^2$

Table 1. The link parameters of the robot manipulator.

$$\theta_1(t) = \arcsin \frac{-l_2 \sin(\theta_2)}{\sqrt{T^2 + H^2}} - \arctan \frac{T}{\sqrt{H^2}}$$

$$\theta_2(t) = \arccos \frac{T^2 + H^2 - l_1^2 - l_2^2}{2 l_1 l_2}$$

$$\theta_3(t) = -\theta_1(t) - \theta_2(t)$$

$$\theta_4(t) + \theta_5(t) = \arccos \frac{n_3}{\sqrt{n_1^2 + n_2^2}} + \arctan \frac{n_1}{n_2}$$

$$\theta_4(t) = \arcsin \frac{T - a_2 \sin(\theta_4 + \theta_5) + a_3 \cos(\theta_4 + \theta_5)}{a_1}$$

$$\theta_6(t) = -0.6t - 0.7,$$

where

$$n_1 = 2a_2 T + 2a_3 H$$

$$n_2 = -2a_3 T + 2a_2 H$$

$$n_3 = a_2^3 + a_3^2 - a_1^2 + T^2 + H^2$$

$$a_1 = \rho_1 l_4 + (\rho_2 + \rho_3) l_2$$

$$a_2 = \rho_2 l_5 + \rho_3 l_1 + \rho_3 b \cos(\theta_6)$$

$$a_3 = -\rho_3 b \sin(\theta_6)$$

$$\rho_1 = \frac{m_4}{m_4 + m_5 + m_6}$$

$$\rho_2 = \frac{m_5}{m_4 + m_5 + m_6}, \rho_3 = \frac{m_6}{m_4 + m_5 + m_6}.$$

REFERENCES

Huang, Q., Yokoi, K., Kajita, S., Kaneko, K., Arai, H., Koyachi, N., et al. (2002). Planning walking

$m_1 = 2.23kg$	$l_1 = 0.332m$	$d_1 = 0.189m$	$I_1 = 3.3kgm^2$
$m_2 = 5.28kg$	$l_2 = 0.302m$	$d_2 = 0.236m$	$I_2 = 3.3kgm^2$
$m_3 = 14.79kg$	$l_3 = 0.486m$	$d_3 = 0.486m$	$I_3 = 3.3kgm^2$
$m_4 = 5.28kg$	$l_4 = 0.302m$	$d_4 = 0.236m$	$I_4 = 3.3kgm^2$
$m_5 = 2.23kg$	$l_5 = 0.332m$	$d_5 = 0.189m$	$I_5 = 3.3kgm^2$
$m_6 = 2.12kg$	$l_6 = 0.290m$	$b = 0.145m$	

Table 2. The link parameters of the biped robot.

patterns for a biped robot. *IEEE Transactions on Robotics and Automation* 17, 280–289

Shih, C. L., Li, Y. Z., Churng, S., and Lee, T. T. (1990). Trajectory synthesis and physical admissibility for a biped robot during the single-support phase. In *IEEE International Conference on Robotics and Automation, 1990. Proceedings*. 1646–1652 vol.3
